# Improvement and extension of anti-EGFR targeting in breast cancer therapy by integration with the Avidin-Nucleic-Acid-Nano-Assemblies

**DOI:** 10.1038/s41467-018-06602-6

**Published:** 2018-10-04

**Authors:** Francesco Roncato, Fatlum Rruga, Elena Porcù, Elisabetta Casarin, Roberto Ronca, Federica Maccarinelli, Nicola Realdon, Giuseppe Basso, Ronen Alon, Giampietro Viola, Margherita Morpurgo

**Affiliations:** 1 Dipartimento di Scienze del Farmaco, Università di Padova, Via Marzolo, 5, 35131 Padova, Italy; 2 Dipartimento di Salute della Donna e del Bambino, Università di Padova, via Giustiniani, 3, 35122 Padova, Italy; 3 ANANAS nanotech, S.r.l., via Altinate 120, 35137 Padova, Italy; 4 Department of Molecular and Translational medicine, University of Brescia, Viale Europa 11, 25123 Brescia, Italy; 5 Department of Immunology, The Weizmann Institute of Science, Rehovot, 7610001 Israel; 6 Present Address: Department of Immunology, The Weizmann Institute of Science, Rehovot, 7610001 Israel; 7 Present Address: Dipartimento di Salute della Donna e del Bambino, Università di Padova, via Giustiniani, 3, 35122 Padova, Italy

## Abstract

Nowadays, personalized cancer therapy relies on small molecules, monoclonal antibodies, or antibody–drug conjugates (ADC). Many nanoparticle (NP)-based drug delivery systems are also actively investigated, but their advantage over ADCs has not been demonstrated yet. Here, using the Avidin-Nucleic-Acid-Nano-Assemblies (ANANAS), a class of polyavidins multifuctionalizable with stoichiometric control, we compare quantitatively anti-EGFR antibody(cetuximab)-targeted NPs to the corresponding ADC. We show that ANANAS tethering of cetuximab promotes a more efficient EGFR-dependent vesicle-mediated internalization. Cetuximab-guided ANANAS carrying doxorubicin are more cytotoxic in vitro and much more potent in vivo than the corresponding ADC, leading to 43% tumor reduction at low drug dosage (0.56 mg/kg). Advantage of cetuximab-guided ANANAS with respect to the ADC goes beyond the increase in drug-to-antibody ratio. Even if further studies are needed, we propose that NP tethering could expand application of the anti-EGFR antibody to a wider number of cancer patients including the KRAS-mutated ones, currently suffering from poor prognosis.

## Abstract (web summary)

The nature of the linker is known to affect the efficacy of antibody–drug conjugate (ADC). Here the authors show cetuximab-guided Avidin-Nucleic-Acid-Nanoassemblies to be superior to cetuximab-doxorubicin conjugate, and show its efficacy in KRAS mutant breast cancer, allowing for therapeutic expansion of anti-EGFR therapy.

## Introduction

Personalized therapy represents the current frontier for the treatment of cancer. Although basic research is untangling the many pathways driving to disease progression and unraveling the details of its hallmarks^1^, new chemical entities/drugs and drug delivery solutions are developed to address or target individual phenotypes. One solution for personalized therapy are the targeted drug delivery systems, namely hybrid entities containing a cytotoxic compound linked to a moiety recognizing an epitope specifically overexpressed at the cancer cell surface. The first successful example of this class are the antibody–drug-conjugates (ADC), which entered in the clinics since 2010^2^, but more complex nanotechnology-based architectures may bring further advantages to the sector in the future.

A limit of ADCs is the limited number of cytotoxic elements that one antibody can carry (drug–antibody ratio; DAR) without losing affinity for its target. In fact, first-generation ADCs that carried clinically approved drugs (e.g., methotrexate, vinblastine, doxorubicin) failed in clinical trials mostly owing to insufficient drug potency^3^. In new generation conjugates, a higher antibody “cytotoxic payload” is achieved either by using more potent cytotoxic compounds (i.e., auristatins, maytansinoids) with IC_50_ in the picomolar range^4^—namely molecules that are too toxic to be used as free ones—or by targeting fast internalizing cancer surface antigens^5^ even if this approach is limited to a restricted number of targeting/antigen combinations. An alternative approach is to use nanoparticle (NP)-based carriers. NPs embed high amounts of drugs and their surface is large enough to host one or more targeting elements, potentially increasing both DAR (the cytotoxic cargo) and avidity for the target.^6–8^ Indeed, many NP-based drug delivery systems are actively investigated, however their advantage over ADCs has not been demonstrated yet, in part because stoichiometric control of NP composition is often complex, making it difficult to carry out quantitative comparisons.

One class of nanoparticles recently described in the literature that allow quantitative control of composition, is the one based on the Avidin-Nucleic-Acid-Nano-Assemblies (ANANAS). ANANAS are nanosized (*Ø* = 120 nm) poly-avidin toroids generated from the condensation of a nucleic acid (NA) filament by the high-affinity interaction with egg-white avidin (1 avidin each 14 ± 4 NA base pairs)^9^, colloidally stabilized by the presence at their surface of a controlled amount of biotinylated poly(ethylene glycol) (PEG)^10^. Thanks to their intact biotin-binding capability, ANANAS have found application as in vitro and in vivo diagnostics tools^11–13^ and have been suggested as potential drug delivery systems^14^ to implement more classic monomeric avidin-based targeted delivery strategies^15^. The high affinity for biotin ligands (Kd 10^−15^ M) permits stoichiometric control of ANANAS composition, in principle allowing investigation on composition/functionality relationships.

In this work, antibody-guided ANANAS were evaluated and quantitatively compared with direct ADCs for targeted drug delivery in breast cancer therapy using an anti-epidermal growth factor receptor (EGFR) antibody (cetuximab) as the targeting element and doxorubicin as the cytotoxic compound. EGFR is an ideal candidate as target cancer epitope as it is often overexpressed in cancer cells and over-expression is associated with advanced disease, poor prognosis, and resistance to therapy^16^. We selected cetuximab (cetux) as the targeting element in order to exploit its ability to bind and promote internalization of EGFR, enabling endocytosis in target cells for intracellular drug delivery. Doxorubicin was selected as the anticancer drug both as a model compound and because it is still widely used as a first line chemotherapeutic agent in many cancer therapies. In addition, a nanoformulated (liposome) doxorubicin with more favorable properties than the free drug is already available in the market (Doxil®), whereas several other examples of nanoformulations have been studied providing a wide literature background for comparative analyses.^5,6,17–20^ Similarly as in ADC, ANANAS drug loading requires the use of a reversible linker chemistry. Lessons learned from early ADCs have shown that the nature of the linker is fundamental in dictating the efficacy of the product^3^. The stability and release mechanism should match, respectively, the product pharmacokinetic profile and its cell fate. Among the different linkers sensitive to the tumor or the intracellular microenvironments described in the literature,^21–23^ here we focused on the acid-reversible hydrazones. In fact, even if early ADCs based on hydrazone linkers failed in the clinics also for their insufficient linker stability (as the case of Mylotarg^2^), we reasoned that the stability requirements are less stringent when using the ANANAS carriers because of their much faster clearance (within 2–6 h)^14^ with respect to the ADCs (days).

The in vitro results show that NP-linked cetuximab internalize EGFR-expressing cells more efficiently than the antibody alone. In vivo, this translates into a more efficient drug delivery system, which is capable to bypass drug resistance in a model of human EGFR positive triple-negative breast cancer.

## Results

### Design and synthesis of nanoformulation building blocks

The ANANAS nanoparticles are essentially “soft” polyavidins. A single NP contains ~ 50 avidins and 1200 biotin-binding sites (BBS) and has ~6000 nm^2^ available for surface functionalization.^11–13^ They can be functionalized with stoichiometric control by simply mixing with biotinylated functional elements at desired biotin:BBS molar ratios and no purification is necessary if neither the BBS become saturated nor the surface area available is exceeded^11–13^. Provided these two requirements are fulfilled, an infinite number of surface composition combinations can be conveniently generated and screened so that, in principle, selection of the best performing composition is relatively easy to perform.

**Fig. 1:**
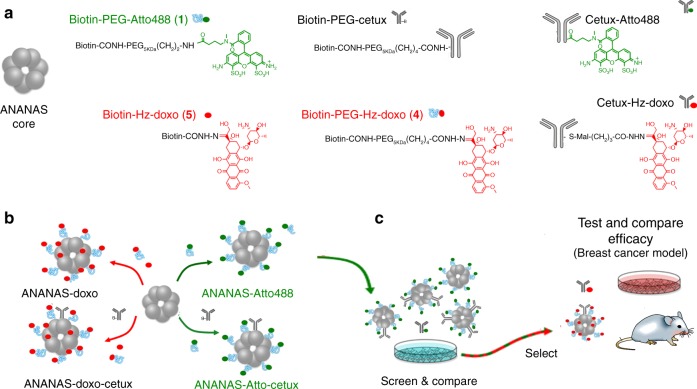
ANANAS assembly and study design. **a** The building blocks for ANANAS functional assembly and the reference antibody conjugates synthesized for this work; **b** ANANAS functional assembly and **c** in vitro and in vivo evaluations

Non-functionalised “core” assemblies were obtained^10,14^ in advance to be decorated (Fig. 1b) with biotinylated functional elements at predetermined biotin:BBS molar ratios. A series of biotinylated elements (Fig. 1, and Supplementary Figures 1 and 3) and were generated, namely biotin-cetuximab, fluorescent biotin-Atto488 and two biotin-doxorubicin conjugates. Atto488 and cetuximab were tethered to biotin through a stable amide bond through a 5KDa PEG spacer (*biotin-PEG-Atto488 and biotin-PEG-cetux)*. In the two biotin-doxorubicin conjugates the drug was linked through an acid-reversible hydrazone bond and the derivatives differ for the length of the spacer between biotin and the drug (no spacer—*biotin-Hz-doxo*—or a 5 KDa PEG—*biotin-PEG-Hz-doxo*). The 5 KDa PEG spacer in both the Atto488 and doxorubicin reagents was selected to maximize the NP PEG surface protection to guarantee long-term colloidal stability^10^, whereas the use of *biotin-Hz-doxo* permits to maximize drug loading by exploiting all of the available BBS, as steric impediments do not permit to saturate all of the available BBS with the bulky PEG reagent^11–13^. Finally, the 5 KDa spacer in biotin-PEG-cetux was selected to guarantee exposure of the targeting moiety at the NP outer surface.

Cetuximab-Atto488 (cetux-Atto488) and cetuximab-Hz-doxorubicin (cetux-Hz-doxo) conjugates were also generated (Supplementary Figure 5) as controls to allow quantitative comparison between direct versus NP-tethered antibody strategies.

### Assembly composition affects cell internalization efficiency

We aimed at evaluating if and to which extent the number of cetuximab/assembly affects its cell targeting ability. Fluorescent (Atto488) labeled cetuximab-linked ANANAS nanoparticles were prepared at three different antibody/NP ratios (10:1, 20:1, 30:1) (Supplementary Figure 8 and Supplementary Table 1) and applied to two breast cancer cell lines representing luminal-type (MCF-7) and basal-type (triple-negative–MDA-MB-231) cancers, respectively. The two cell lines also differ for the expression levels of EGFR (MDA-MB-231 ~ 20-fold higher than MCF-7), as also confirmed experimentally (Supplementary Table 4). MDA-MB-231 also carries a KRAS mutation, which contributes to its malignity and resistance therapies targeting the EGFR-dependent transduction cascade^16^. The Atto488 dye was selected as a probe because of its high fluorescence brilliance, which is not influenced by the local pH. In this way, also NPs internalized through the low pH endosome–lysosome pathway are detectable in fluorescence based assays.

**Fig. 2:**
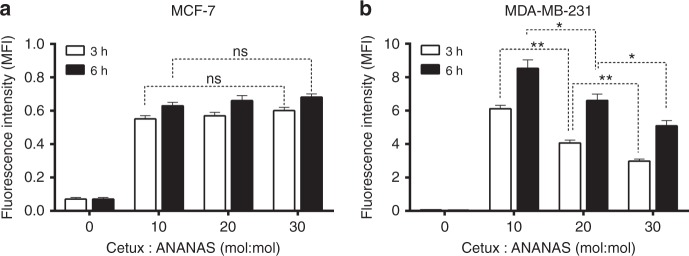
Cell targeting by formulations with different Cetux/ANANAS molar ratio. Fluorescence intensity associated to MCF-7 or MDA-MB-231 cells after 3 and 6 h incubation with ANANAS/Atto488 formulations (7.5 μg/mL) at different Cetuximab:ANANAS molar ratio. Median fluorescence intensity (MFI) values were normalized for the ANANAS-Atto488 loadings (Supplementary Table 1). Data presented as mean ± SEM of three independent experiments (each in triplicate): *p < 0.05, **p < 0.01 (paired *t* test)

The amount of fluorescence (Fig. 2 and Supplementary Figure 9) associated with the MDA-MB-231 cells measured by FACS analysis was higher (between 6–10-fold) than that associated with MCF-7 as expected from their different EGFR expression level (Supplementary Table 4).

Interestingly, cetuximab:ANANAS molar ratio affects NP cell internalization efficiency in overexpressing but not in normally expressing EGFR cells: in MCF-7 cells, no significant difference in the levels of cell-associated fluorescence was observed with the number of cetuximab/nanoassembly. On the contrary, with the MDA-MB-231 cell line, an inverse correlation between cetuximab:NP load and cell-associated fluorescence was obtained, the most efficient formulation being the one carrying 10 antibodies/NP (ANANAS-Atto488-cetux10) with a surface density of 600 nm^2^ every cetuximab molecule. This inverse correlation can be explained by hypothesizing a receptor saturation or cross-linking phenomenon upon interaction with the NP displaying high cetuximab surface concentration, which could impair NP internalization especially in the high EGFR-expressing cells. Interestingly, the trend observed is opposite to what described with another anti-EGFR-targeted nanoformulation, namely anti-EGFR Fab-targeted immunoliposomes^24^, for which similar surface densities of the targeting element as the ones used here were probed. The different chemical nature (and mechanical properties, fluidity) of the two nanosystems could be responsible for a different internalization pathway after docking to the receptor. Based on these results, a Cetux:NP molar ratio of 10:1 was selected for all subsequent investigations.

### Cetuximab trafficking in EGFR+cell is affected by its cargo

**Fig. 3:**
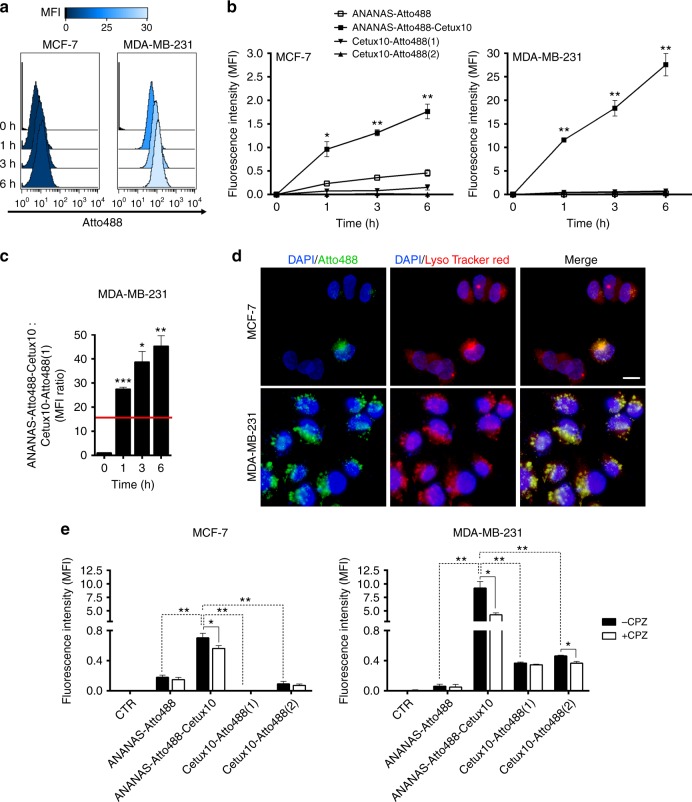
Cell targeting and internalization of Atto488 carrying formulations. Representative histograms **a** and quantitative analysis **b** of MFI associated with MCF-7 and MDA-MB-231 cells upon incubation with ANANAS-Atto488 (Atto488 3.5 × 10^−9^ M), ANANAS-Atto488-Cetux10 (cetuximab 0.58 μg/mL, Atto488 3.5 × 10^−^^9 ^M) and cetux-Atto488 at low and high concentration (Cetux-Atto488(1): same Cetux as in ANANAS-Atto488-Cetux10; Cetux-Atto488(2): same Atto488 as in ANANAS-Atto488-Cetux10; MFI values have been normalized for the Atto488 brilliance in each formulation (40% and 61% of that of the free dye when tethered to ANANAS or to cetuximab, respectively); **c** Ratio between cell-associated MFI in MDA-MB-231 when treated with ANANAS-Atto488-Cetux10 and Cetux-Atto488(1) at the same cetuximab concentration (0.58 μg/mL). The horizontal red bar indicates the ratio between FAR ANANAS-Atto488-Cetux10 and FAR Cetux-Atto488 and corresponds to the maximum enhancement factor expected for the ANANAS-based carrier in case of a pure FAR improvement mechanism. A similar trend was observed with the MCF-7 cells. However, owing to the low fluorescence registered in the cetux-Atto488(1)-treated samples, the actual MFI ratio—which can only be estimated—was not reported in the figure. (The original data set is reported in Supplementary Table 6). Data presented as mean of *n* = 3 experiments (each in triplicate) ± SEM. Paired *t* test of treated samples respect to time 0: *p < 0.05, **p < 0.01, ***p < 0.001. **d** Fluorescence microscopy imaging of MCF-7 and MDA-MB-231 after 6 h treatment with ANANAS-Atto488-Cetux10. Green: Atto488, red; Lysotracker, blue channel nuclei staining with DAPI. × 60 magnification, size bar = 20 µm. **e** Effect of the endocytic inhibitor chlorpromazine (CPZ) on ANANAS internalization: MFI associated with MCF-7 and MDA-MB-231 cells pre-treated with CPZ 30 µM, and then treated for 6 h with the different Atto488 formulations. Data presented as mean of three independent experiments (each in triplicate) ± SEM (paired *t* test): *p < 0.05, **p < 0.01. For the concentrations of Atto488-loaded reagents, see above

Cetuximab-targeted ANANAS were quantitatively compared with cetuximab and to untargeted ANANAS for their ability to bring their cargo to the EGFR-expressing cells. In both MCF-7 and MDA-MB-231, the fluorescence signal associated with cells treated with ANANAS-Atto488-cetux10 was dramatically higher than the one induced by untargeted ANANAS-Atto488 or by the antibody conjugate (Fig. 3a, b and Supplementary Figure 9), confirming NP-specific targeting. Notably, at any time point tested, the fluorescence gain of NP-treated cells with respect to the same cells treated with cetux-Atto488 at the same antibody concentration is beyond the one expected from the two formulations relative fluorescence-to-antibody ratio (Fig. 3c and Supplementary Table 6). This indicates that internalization of cetuximab-guided NPs occurs, at least in part, through a distinct and more rapid internalization pathway than that of free cetuximab. The notion that the two systems adopt different internalization pathways is also supported by the results obtained in the presence of chlorpromazine (CPZ). This inhibitor of clathrin-mediated endocytosis reduced the uptake of the NP-antibody conjugate but not the internalization of the free antibody incubated at equimolar concentration (Fig. 3e, Supplementary Table 5).

It is known that internalization of EGFR can occur through either lipid-raft or clathrin-mediated pathways and different stimuli can promote a switch from one pathway to the other. For example, stimulation with low doses of epidermal growth factor (EGF) leads to receptor internalization via clathrin-mediated endocytosis, whereas high doses induce the raft-dependent pathway^25^. Cetuximab, a competitor of the EGF/EGFR interaction, is reported to promote the raft-mediated EGF internalization, in line with our data in which CPZ had limited impact on cetux-Atto488 internalization. On the other hand, it was also shown that when a switch from lipid-raft to clathrin-mediated pathways is promoted by the presence of the cholesterol–sequestrating drug nystatin, cetuximab-mediated internalization is accelerated^26^. Our data suggest that, at least in part, NP tethering of cetuximab promotes the clathrin-mediated mechanism and, similarly to nystatin, it accelerates internalization. Indeed, the addition of nystatin (Supplementary Figure 10), did not generate any significant effect on cetux-guided NP cell internalization. Independently of the mechanism involved, the all nanoparticles end-up into acidic endosomes, as shown by immunofluorescence NP visualization in the presence of the late endosome marker lysotracker-red (Fig. 3d).

**Fig. 4:**
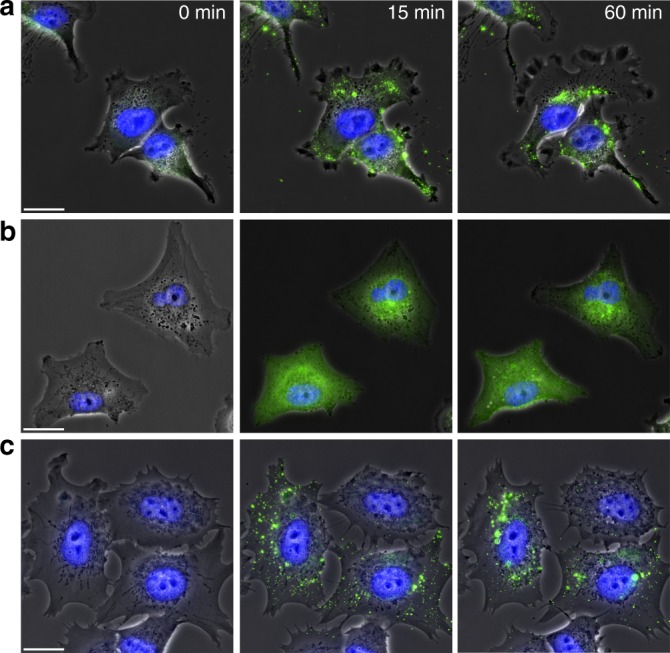
Dynamics of NP internalization. Extracts of live fluorescence microscopy images of **a** and **b** MDA-MB-231, and **c** MCF-7 cells exposed to: **a** and **c** ANANAS-Atto488-cetux10 (15 µg/mL, Atto488 = 1.6 × 10^−7^ M) or **b** cetux-Atto488 (15.1 μg/mL, Atto4888 1.4 × 10^−7^ M) for 10 min at 37 °C in a flow chamber, and then subjected to washing in order to remove unbound nanoparticles. Cells were continuously subjected to low shear flow (0.02 dyn/cm^2^) and imaged for up to 60 min thereafter. The panels display the superimposed images from the Atto488-related signal (green), the phase contrast and nuclei related one (blue, Hoescht). Size bar 20 µm. Note that during the imaging period the majority of the Atto488-associated nanoparticles move from the cell periphery to the perinuclear region, whereas the majority of the fluorescent signal associated to cetux-Atto48 remains at the cell surface. The entire live imaging videos from which these representative images were extracted are available in Supplementary Movie 1 and Supplementary Movie 2 (Supplementary Movie 3 is for the non-targeted Formulations; Supplementary Movie 4 and Supplementary Movie 5 show the trafficking process of targeted formulation at longer times- between 1 h to 6 h)

The dynamics of atto488antibody conjugate and NP-trafficking inside the various target cells was further investigated through a series of time-lapse fluorescence microscopy experiments (Fig. 4 and Supplementary Movies 1–6) in which we followed the fate of the NPs following exposure to cells. These experiments were carried out in a flow chamber to allow rapid exchange of the perfused solution containing the fluorescent sample with drug-free medium. In this way, the time of contact between the cells and the fluorescent sample was restricted to 10 min and the very first cohort of nanoparticles interacting with multiple cells was tracked in real time. In both cell lines ANANAS-Atto488-cetux10 particles, but not the antibody-free assembly are taken up and concentrate into endosomal and lysosomal compartments, which concentrate in the perinuclear region ~ 1 h after docking. This is more evident for the MDA-MD-231 in which the number of NP containing vesicles is also greater than in the MCF-7 in line with the FACS data (Fig. 3). Notably, no fluorescent signal was lost during the 6 h experimental time limit period suggesting that all of the NPs and their cargo remain stably entrapped inside the cells once internalized (Supplementary Figure 7). This is a favorable property and the main aim of our original nanoassembly design. The time-lapse experiment also strengthens the hypothesis of the different internalization between NP and antibody conjugate. In fact, although the fluorescent signal generated by the NPs clusters in fast-trafficking vesicle compartments, the majority of the antibody conjugate remains at the cell surface 1 h after docking.

### Doxorubicin is released from ANANAS only upon lysosome entrance

An ideal drug delivery system should fulfill a series of requirements: it should (a) be colloidally stable, (b) retain its cargo when in non-target districts (e.g., serum), and (c) release it promptly upon reaching its desired destination. Here, we also aimed at guarantee ease of assembly and maximal drug loading through the exploitation of all the available BBS. As to the linker between the biotin element and the drug, a reversible chemistry is necessary to permit release of the active cargo similarly as in ADC. Here we focused on the acid-reversible hydrazones, which display multiple advantages: (a) their sensitivity to mild acidic pH fits well the nanoparticles’ lysosome-endosome internalization pathway; (b) they are easy to obtain; and (c) being equilibrium-based bonds, upon hydrolysis they return the drug in its original form and this is an advantage in case of potential regulatory requirements.

Drug-loaded ANANAS (Supplementary Table 2) were generated in one pot solution using together the short and PEG spaced biotin-Hz-doxorubicin conjugates (Fig. 1) by mixing them with the core ANANAS at desired molar ratios. Up to 650 doxorubicin molecules/NP were stably tethered whereas no free doxorubicin nor any other biotin conjugate was present in the assembly solution as demonstrated by gel permeation chromatography (Supplementary Figure 8B).

**Fig. 5:**
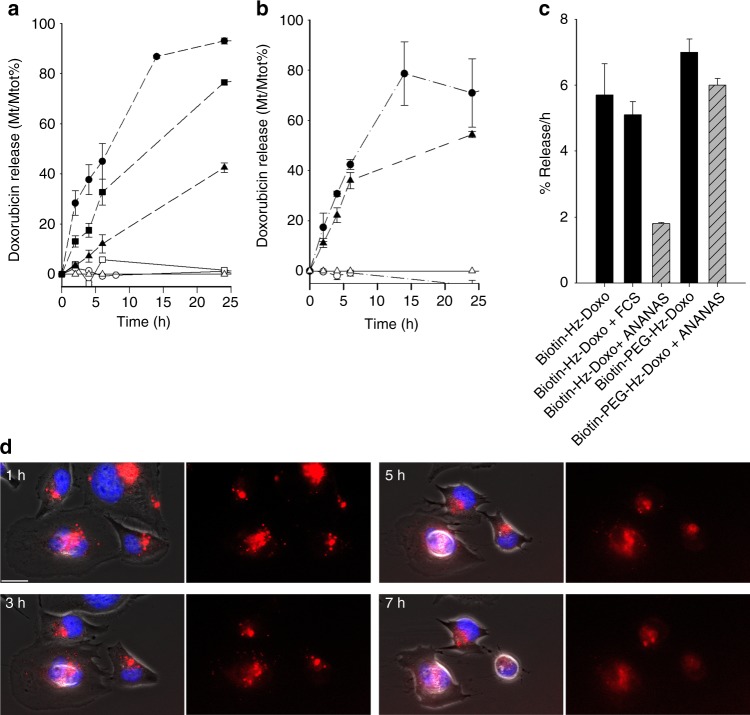
Drug release in neutral and acidic buffers and upon cell internalization. Kinetics of doxorubicin release from **a** biotin-Hz-doxorubicin, **b** biotin-PEG-Hz-doxorubicin at pH 7.4 (open symbols) or 5.0 (full symbols) in protein-free buffers (circle symbols), and in presence of 10% fetal calf serum (square symbols) or ANANAS (triangle symbols). **c** Hourly release rates (see also Supplementary Table 3) extrapolated from the linear part of curves in **a** and **b**; Data were generated at 37 °C and the results are expressed as % of total release. Release experiments were carried out in duplicates and data are presented as the mean ± standard deviation (S.D). **d** Extracts of live fluorescence microscopy images of MDA-MB-231 cells exposed to ANANAS-Hz-doxorubicin-cetux10 (36 µg/mL) for 1-h at 37 °C, then fluxed in flow a chamber to remove unbound nanoparticles and imaged for up to 7 h; the right panels show the doxorubicin-related (red) signal, the corresponding left panels show the red doxorubicin images superimposed with the phase contrast and nuclei (blue, Hoechst) ones. Size bar 20 μm. Along the experiment, the doxorubicin-related red signal is initially concentrated in cytoplasmic vesicles compartments, then it starts diffusing out from there and concentrating in the cell nuclei. The entire live imaging Video is available in Supplementary Movie 6

In vitro release experiments (Fig. 5a–c, Supplementary Table 3) showed that in the lysosome-mimicking acidic conditions doxorubicin is released from the nanoparticles within 16–50 h, whereas in the neutral serum-mimicking situation the hydrazone bond is indefinitely stable. At neutral pH, the two biotin conjugates are stable both as free molecules and when tethered to the ANANAS surface. At pH 5.0, the drug is released from the two conjugates in ~ 8–10 h, in line with literature documented hydrazone-doxorubicin hydrolysis data^22,27–29^ and independently from the presence or absence of the 5KDa PEG spacer. However, when tethered to ANANAS, the short linked hydrazone (but not the PEG spaced) displays significantly greater resistance to hydrolysis (*t*/2 = 28 h), suggesting the existence of a local buffering microenvironment in proximity of the NP protein surface. This difference supports previous evidence^27–30^ that the local environment or the nature of linker influences hydrazone stability. In this case, we hypothesize that lysine 111, which is located in proximity to the entrance of the biotin-binding pocket^31,32^, may be responsible for a local buffering effect. Despite the ANANAS-related higher hydrazone hydrolytic stability, live fluorescence microscopy imaging showed that doxorubicin is released from the ANANAS-Doxo-cetux10 assembly upon its entrance in the cells and it is capable to reach the nuclei (Fig. 5d and related Supplementary Movie 6).

### Cytotoxicity of cetuximab-ANANAS-doxo vs cetux-Hz-doxo

**Fig. 6:**
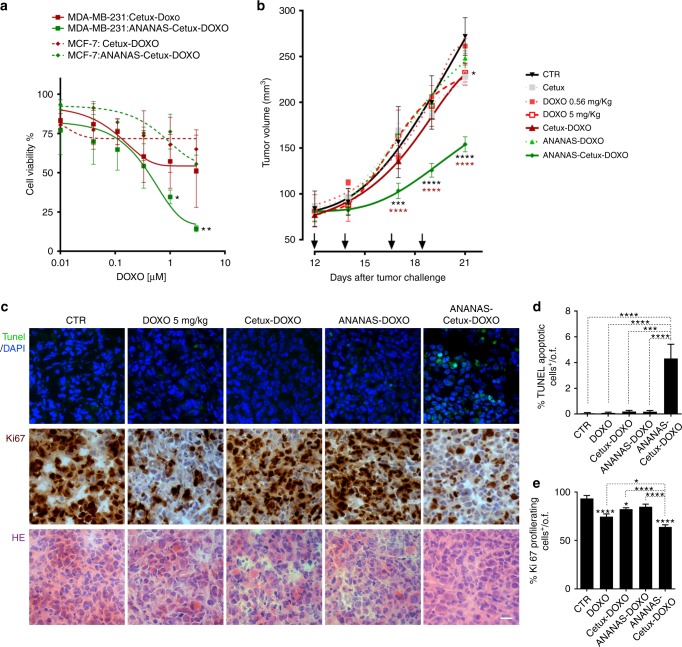
In vitro and in vivo activity of doxorubicin carrying formulations. **a** Cell viability was assessed in MCF-7 and MDA-MB-231 cell lines after 6 h treatment and wash out for following 72 h (MTT test). Data presented as mean of *n* = 2 experiments ± SEM (paired *t* student) *p < 0.05, **p < 0.01; **b**. Tumor volume measured in xenografts of MDA-MB-231 cells injected subcutaneously in the flank of NOD/SCID mice (*n* = 6/group). Animals were treated four times (as indicated by the arrows) with the different doxorubicin containing formulations (doxorubicin low = 0.56 mg/kg), free cetuximab (same dosage as in ANANAS-doxo-cetux10, corresponding to 2.8 mg/kg—0.92 mg/m^2^—in cetuximab) or doxorubicin high dose (5 mg/kg) by i.v. injection, and killed at day 21. Data are presented as mean ± SEM (two-way ANOVA ANANAS-cetux-doxo against ctrl: black—upper asterisks or against cetux-doxo: brown - lower asterisks): *p < 0.05, **p < 0.01, ***p < 0.001, ****p < 0.0001. **c** Histological analyses of excised tumors, stained for TUNEL, Ki67, and hematoxylin/eosin (HE). × 40 magnification, size bar = 25 µm. **d**, **e** TUNEL and Ki67 quantification represented as the percentage of positive-cells over the total number of nuclei per optical field (o.f.). Data are presented as mean ± SEM (paired *t* test) *p < 0.05, **p < 0.01, ***p < 0.001, ****p < 0.0001

The in vitro cytotoxicity of ANANAS-doxo-cetux10 was tested in both MCF-7 and MDA-MB-231 cells in parallel with the cetux-Hz-doxo conjugate, the non-targeted formulation and free doxorubicin. Cetuximab-targeted ANANAS was more cytotoxic than the antibody conjugate in both cell lines (Fig. 6 and Supplementary Figure 11 A–B) supporting the evidence (Fig. 3C) that ANANAS tethered cetuximab internalizes the cells more efficient than the antibody alone. In order to evaluate if our formulations are toxic in normal cells we used a non-tumoral epithelioid human cell line (HEK-293) that expresses low level of EGFR^33^, in which ANANAS formulation showed no significant activity (Supplementary Figure 11C) suggesting a certain preference toward EGFR-overexpressing cancer cells.

The cetux-targeted nanoformulation was more effective in MDA-MB-231 than in MCF-7 (IC_50_ 0.1 and 4 µM, respectively) confirming that cytoxicity is strongly dependent on the ability of the assemblies to internalize the cells. Notably, in the MDA-MB-231 cells the NP formulation was even more effective than the free drug (IC_50_ = 1 µM). This was surprising taking into consideration the fact that the nanosystem needs to undergo the internalization/drug release steps, whereas the free drug can diffuse freely from the medium. It is also interesting to note that cetuximab alone, used at the same concentration as the cetux-Hz-doxo conjugate has no cytotoxicity indicating that inhibition of the EGFR-signaling cascade is not responsible for the effects observed. Indeed, MDA-MB-231, which is a KRAS mutant^21,34^ should not respond to cetuximab therapy.

### ANANAS-doxo-cetux10 efficacy in vivo at low drug dosage

The results of the in vivo studies are highly encouraging (Fig. 6b–e). As the main purpose of this work was to test the hypothesis that antibody-guided ANANAS can be more effective than ADC and this higher efficacy could be exploited to reduce the amount of cytotoxic compound administered to the patient, we purposely selected a low-drug dosage (0.56 mg/kg), namely 10-fold lower than the one classically used for in vivo testing of doxorubicin nanodelivery systems.^17–20,35^ Considering the strong correlation observed in vitro between internalization/cytoxicity and the expression of the EGFR receptor, in vivo evaluation was carried out in the MDA-MB-231 mouse xenograft.

Tumor-bearing mice were treated with four injections of ANANAs-doxo-Cetux10 (total 2.24 mg/Kg) and a significant time-dependent reduction of tumor burden (reduction of tumor mass of 43%) was observed with a statistical significance versus all control groups (including free doxorubicin, cetuximab-doxorubicin conjugate and ANANAs-doxo groups). Accordingly, immunohistochemical analysis of the explanted tumors showed a significant reduction of proliferating/Ki67^+^ cells, and an increase of apoptotic/TUNEL^+^ areas (Fig. 6c–e). No sign of toxicity was observed nor any decrease in animal body weight (Supplementary Figure 12) in all treated groups with the exception of high dose (5 mg/kg) doxorubicin, where a mild tumor reduction (together with a mild reduction in proliferation but no apoptotic effect) was associated with heavy toxicity (four animals out of six died during the experimental time with a median survival time of 18 days) and weight loss. The fact that the high free doxorubicin dosage lead to reduced proliferation without affecting cell death is in line with the hypothesis that the ANANAS-cetux-Doxo formulation is capable to bring higher intracellular doxo concentration than the administration of the free drug. Indeed, the effect of doxorubicin on tumor cells (anti-proliferation vs pro-apoptosis) has been shown to depend on the drug concentration,^36^ namely at low concentration the drug promotes cell senescence (thus stops proliferation), whereas at high doses it can induce cell apoptosis.

Interestingly, this result is similar to the one observed for cetuximab-Hz-doxorubicin when administered in combination with nystatin, an enhancer of cell internalization^26^. This further support that the higher efficacy here obtained is related to increased internalization of cetuximab promoted by ANANAS tethering.

## Discussion

The possibility to tailor assembly composition and surface properties enabled us to demonstrate that the advantage of antibody-guided ANANAS with respect to an antibody conjugate goes beyond what expected from a simple increase in the DAR. Cetuximab-guided cell internalization is accelerated by ANANAS tethering and triggers, at least in part, a different and more efficient internalization pathway. Although the reason for this should be further explored, this is likely the reason why the ANANAS-based formulation is more effective than the antibody conjugate in vivo despite the same doxorubicin dose and the same chemistry (hydrazone) for doxorubicin tethering.

Our data also show that surface composition in nanoparticles matters. Relatively minor changes in the targeting element surface density greatly affect NP behavior, even if this effect varies also with the nature of the cell system adopted for investigation. Although the same rules may not apply to different nanosystems (e.g., liposomes vs solid or protein-based nanoparticles), it is clear that surface composition needs to be quantitatively tuned in order to identify the most performing formulation for the desired application and then monitored to obtain consistent results. This is a potential drawback for nano-based delivery systems with respect to ADCs for which fewer variables need to be monitored. A potential consequence could be that development costs of NP-based systems may be larger. Still, the advent of new in vitro and ex vivo screening technologies, including the use of 3D cell cultures and organoid systems, could help reducing this gap.

As a general conclusion, the ANANAS platform is a promising tool for targeted cancer therapy. The in vivo results show therapeutic efficacy at a dose of doxorubicin that is ~ 10-fold less than the one classically used in preclinical studies with other nano-based doxorubicin carriers and a cetuximab dose that is well below the one that is normally used in cetuximab-based therapy. It is important to point out that the role of cetuximab in the assembly is related to its EGFR targeting/binding property and it is not dependent on its effects on the EGFR-related transduction pathway. For this reason, the therapeutic effect is achieved on a tumor with poor therapeutic options and that is resistant to cetuximab or doxorubicin treatment as such.^37^ As a consequence, application of this antibody—when combined to ANANAS–could potentially be extended to a wider number of breast cancers, including the KRAS-mutated ones, as well as to other cancer types expressing EGFR, regardless from the “first line” efficacy of cetuximab alone.

Finally, further advantage of the ANANAS platform relates to its high versatility which permits to vary both cargo and targeting element without the need for complex chemistries. The possibility to combine fluorescent labeling with drug loading in a single formulation or to use alternatively fluorescent or drug-loaded analog assemblies as in this work could bring new theranostic solutions for personalized cancer therapy in the future. Still many issues remain to be investigated. Future work will extend evaluation of this approach to a wider number of cancers using different combinations of targeting elements and cytotoxic cargoes, including the most cytotoxic compounds used in new generation ADCs, to further reduce the toxicity risks currently associated with this class of targeted drug delivery systems. In addition, even if ANANAS have shown to be poorly immunogenic in animal models, the role of the immune-system along the use of these formulations should be further investigated in order to fully understand their potential in clinical settings.

## Methods

### Reagents and instrumentation

Biotin-hydrazide (Ez-Link^TM^, Thermo Scientific, 21339); biotin-PEG_5KDa_-SVA (biotin-PEG-SVA) and biotin-(PEG_5KDa_)-NH_2_ (biotin-PEG_-_NH_2_) were from Laysan Bio (Huntsville, AL, USA); Atto488 *N*-hydroxysuccinimidylester (Atto488-NHS, ATTO-TEC, gmbh, AD 488-3, Siegen, Germany); Cetuximab (Erbitux^®^, MerckSerono, 5 mg/mL). Absolute ethanol, diethyl ether (Et_2_O), and glacial acetic (AcOH) acid were from Carlo Erba reagents, (Italy). Ethyl acetate (EtOAc) AnalaR Normapur was supplied from WVR (Radnor, Pennsylvania, US). Ninhydrin (cat no. 415720750) and triethylamine (TEA, cat no. 15791) were obtained from Acros Organics); NHS was from ABCR, cat. no. AB110699); 2-(4-hydroxyphenylazo)benzoic acid (cat. no. 54793) and *N*,*N*’-dicyclohexylcarbodiimide (DCCI, cat. no. 36650), *tert*-butyl carbazate (BOC-Hz, cat no. B91005); 2,4,6-trinitrobenzenesulphonic acid (cat no. 92823); 2-iminothiolane hydrochloride (Traut’s reagent, cat no. I-6256); 6-maleimidohexanoic acid (6MC, cat no. 755842); doxorubicin hydrochloride (European Pharmacopoeia Reference Standard); trifluoroacetic acid (TFA, cat. no. T6508), acetonitrile (ACN), dichloromethane (DCM), dimethylformamide, and dimethyl sulfoxide (DMSO) and all other reagents were obtained from Sigma-Aldrich (Italy). All water used was of double-distilled (dd-H_2_O) grade. Gravity NAP10 disposable columns prepacked with Sephadex G-25 DNA Grade were from GE Healthcare. Spectroscopic analyses were conducted with a Varian Cary^®^ 50 UV-Vis spectrophotometer. Fluorescence was determined with a Jasco FP-6200 spectrofluorimeter. Nuclear magnetic resonance (NMR) analyses were made with Bruker AMX 300 and 400 MHz NMR spectrometers; NMR spectra were analyzed with Bruker’s TopSpin^TM^ software. FPLC (fast protein liquid cromatography), analyses were conducted with an AKTA Purifier FPLC system (GE-healthcase) associated with a Waters 2414 RI (refractive index detector). Different size exclusion columns were used: Superdex Peptide 10/300 GL, Superose 6 10/300 GL, Sepharose 6 FF (GE Healthcare). Reverse Phase-High Performance Liquid Chromatography (HPLC) analyses were performed on an Agilent Chromatography system (USA), model 1220 Infinity LC equipped with diode array detector. NP size measurements were carried out by dynamic light scattering using the Zetasizer Nano ZS (Malvern, Malvern UK). The mass of polyethylene glycols compounds was measured using the AB SCIEX 4800 Matrix Assisted Laser Desorption Ionization Time Of Flight (MALDI TOF)/TOF™ Analyzer (SCIEX, Toronto, Canada). For detecting the mass of low molecular weight compounds, we employed the XEVO G2-S Electrospray Time of Flight (ESI-TOF) mass spectrometer (Waters, Milford, MA, USA).

### Synthesis of biotin conjugates

Biotin-PEG_5KDa_-Atto488 (biotin-PEG-Atto488, compound 1) was obtained (Supplementary Figure 1) by the reaction of biotin-PEG_-_NH_2_ with 1.3 equivalents of Atto488-NHS in 10 mM Phosphate, 150 mM NaCl,pH 7.4 (PBS) buffer and purified from excess unreacted dye by extensive dialysis (cutoff 10.KDa) against dd-H2O.

Biotin-PEG_5KDa_-valeric ω-(doxorubicin 13-acylhydrazone) (biotin-PEG-Hz-doxo, compound 4) was obtained through a multi-step synthesis (Supplementary Figure 1). In the first step, biotin-PEG5K-succinimidyl valerate was converted into α-biotin, ω-NH-BOC-valeric hydrazide-PEG_5KDa_ (compound 2) reacted BOC-Hz (1 equivalent) in the presence of TEA (1 equivalent). in anhydrous mixture of DCM/EtOAc (1:1). After overnight reaction, the product was precipitated with cold Et_2_O, recovered by gooch filtration and dried in vacuo, then purified by hot-cold crystallization in EtOAc and dried in vacuo. Quantitative conversion compound 2 was confirmed by ^1^H-NMR analysis. (^1^H-NMR (CDCl_3_, 300 MHz) of biotin-PEG5K- valeric BOC-hydrazide: δ4.49 and 4.32 ppm (m, 2 H, -NH-CHR-CHR-NH- in biotin ring). δ3.64 ppm (m, ~ 454 H, -(CH_2_CH_2_-O)_n_- in PEG_5000_ chain). δ1.459 (s, 9 H, -O-(CH3)_3_ BOC group). The BOC group was removed in 95% TFA (45 min) and α-biotin, ω-valeric hydrazide-PEG_5KDa_ (compound 3) was isolated as solid by precipitation and extensive washing with cold dry Et_2_O. Full BOC removal was confirmed by ^1^H-NMR analysis. The product was also characterized by MALDI TOF mass spectroscopy. ^1^H-NMR (CDCl_3_, 300 MHz) of biotin-PEG5K- valeric hydrazide: δ4.49 and 4.32 ppm (m, 2 H, -NH-CHR-CHR-NH- in biotin ring). δ3.64 ppm (m, about 454 H, -(CH_2_CH_2_-O)_n_- in PEG_5000_ chain) (Supplementary Figure 2). Finally, compound 3 was mixed with 3.2 equivalents of doxorubicin in anhydrous DMSO containing 5% AcOH at a final PEG concentration of 180 mg/mL. After 40 h, the mixture was precipitated in dry Et_2_O and the PEG containing fraction was extracted (5 ×) from the solid mixture with dry DCM. The collected organic fractions were filtered and evaporated under vacuo. Product (biotin-PEG-Hz-doxo, compound 4) identity was confirmed by ^1^H-NMR and MALDI (Supplementary Figure 2), the absence of unconjugated doxorubicin was confirmed by gel permeation chromatography (Superose column and PBS as the eluent) (biotin-PEG-Hz-doxo, Supplementary Figure 8B). The doxorubicin:biotin-PEG ratio (0.44:1) in the product was calculated by titrating the PEG and doxorubicin contents by iodine assay^38^ and UV-Vis analysis, respectively.

Biotin (doxorubicin 13-acylhydrazone) (biotin-Hz-doxo, compound 5) was obtained at room temperature (RT) by mixing biotin-hydrazide (Ez-Link™ Hydrazide-Biotin, Thermo scientific) with 1 equivalent of doxorubicin in anhydrous DMSO containing 5% of acetic acid at a final concentration of 15 mg/mL. After 3 days, the product was isolated by precipitation in cold dry Et_2_O and dried in vacuo. Product identity (doxorubicin: biotin = 0.88) was confirmed by ^1^H-NMR and ESI-TOF mass spectrometry (Supplementary Figure 3).

### Synthesis of cetuximab derivatives

The strategy for all cetuximab conjugate synthesis is summarized in Supplementary Figure 5.

Biotin-PEG_5KDa_-cetuximab (biotin-PEG-cetux) was obtained by reacting cetuximab (2 mg/mL in 0.1 M borate buffer, pH 8.5) with 20 equivalents of biotin-PEG-SVA for 2 h at RT. The conjugate was purified from reaction by-products by ultrafiltration (cutoff 30 KDa) using a 50 mL Amicon ultrafiltration unit, using PBS as the exchange buffer. PEG^38^, Biotin^39^, and cetuximab contents (*E*_0.1%, 1 cm_ = 1.45) in solution were used to estimate the biotin-PEG:Ab ratio (1.5:1) in the product.

Cetuximab-Atto488 was obtained by the reaction of cetuximab (2 mg/mL in 0.1 M borate buffer, pH 8.5) with five equivalents of Atto488-NHS for 2 h at RT, purified from low molecular weight (MW) side-products gel filtration (NAP10, GE healthcare), followed by dialysis (cutoff 10 KDa) against PBS. The degree of antibody modification (1.4 Atto/cetuximab) was calculated from the ratio of Atto488 dye and antibody concentrations measured by UV-Vis spectroscopy (Supplementary Figure 6A), taking into consideration their molar absorptivities (Atto488 *ε*_501nm_ = 9 × 10^4^; Cetuximab *E*_280nm 0.1%, 1cm_ = 1.45). The ability of the conjugate to recognize cell-associated EGFR was confirmed by flow cytometry (Supplementary Figure 7).

Cetuximab-6MC-Hz-Doxorubicin (cetuximab-Hz-doxo) was obtained according to the literature^40,41^. In brief, cetuximab (2 mg/mL in 0.1 M borate, pH 8.5) was added of a sixfold molar excess of 2-iminothiolane hydrochloride (Traut’s reagent)^42^; After 1 h (RT) the conjugate was purified by gel permeation (NAP10 column), using 10 mM acetate, 5 mM EDTA pH 6.0 eluent, then immediately added of 10-fold molar excess of 6MC-Hz-Doxorubicin^40,41^ (Supplementary Method 1 and Supplementary Figure 4). After 3 h at RT, the conjugate (cetuximab-Hz-doxo) was separated from low MW by-products by gel filtration (NAP10 column) using PBS as eluent. The final drug–antibody ratio (DAR = 1.8) was calculated from the UV-Vis spectrum (Supplementary Figure 6B). The ability of the conjugate to recognize cell-associated EGFR was confirmed by Flow cytometry (Supplementary Figure 7)

### Preparation and characterization of ANANAS formulations

A core ANANAS preparation containing the minimum amount of 5 KD methoxy-PEG (12.5% BBS), to guarantee buffer solubility^10^ was kindly provided in freeze dried (from a 0.05% tween 20 and 1% D-trehalose buffer solution) by ANANAS nanotech S.r.l (cat. number N_0101). For the preparation of ATTO488 fluorescent ANANAS (Supplementary Table 1), core NPs were reconstituted in PBS buffer at desired concentration by adding biotin-PEG-cetux at the desired molar ratio (cetux: NP between 0 and 30), followed by biotin-PEG-ATTO488 at 3:10 biotin:BBS molar ratio. Assemblies were used without any further purification. Atto488 tethering to the NP was verified by size exclusion chromatography (Supplementary Figure 8A). Formulations were analyzed for size, zeta-potential (Malvern zetasizer), and UV-Vis properties (Cary50). The number of Atto488 molecules attached to the particles was measured from the UV-Vis spectra (Supplementary Figure 8C), on the basis of the 280 nm (avidin, DNA, and Atto488) and 501 nm (Atto488 only) absorption values

Doxorubicin-loaded ANANAS (Supplementary Table 2) were generated at concentrations between 0.1 and 2.7 mg/mL mixing the core NPs with the different biotin derivatives (biotin-PEG-Hz-doxo, biotin-Hz-Doxo and biotin-cetux) at predefined molar ratios. The absence of unbound biotin derivatives in the final mixtures was verified by size exclusion chromatography (Supplementary Figure 8B). All formulations were analyzed for size, zeta-potential (Malvern Zetasizer) and UV-Vis properties (Varian Cary50). The number of doxorubicin molecules attached to the particles was measured from the UV-Vis spectra (Supplementary Figure 8D), on the basis of the 280 nm (avidin, DNA and doxorubicin) and 480 nm (Doxo only) absorption values.

### Doxorubicin release studies

Doxorubicin release from conjugates 4 and 5—as free molecules or when tethered to ANANAS—was evaluated at three pHs (100 mM phosphate pH 7.0, 100 mM phosphate, pH 6.0 and 100 mM NaAcetate, pH 5.0), in the presence or absence of 10% fetal calf serum (FCS). Samples (80 µM in doxorubicin) were incubated at 37 °C in the desired solution and at selected time-points the concentration of free doxorubicin in solution was measured by HPLC (Phenomenex Kinetex C18, 5 µm, 4.6 × 250 mm, 100 Å; eluant A: 10 mM NH4OAc, pH 5.5, eluant B 5% A in ACN; gradient from 5% A to 90% B in 40 min, chromatogram readout at 480 nm) or UV-Vis. A preliminary salting out (75% saturated (NH_4_)_2_SO_4_) or cold acetone precipitation step was performed on samples containing FCS and/or NPs prior to UV-Vis or HPLC analysis respectively to remove the unwanted protein fraction.

### Cell lines

Human breast adenocarcinoma cells MCF-7 (purchased from ATCC, cat no. HTB-22) and MDA-MB-231 (provided by Dr. R. Giavazzi, Istituto M. Negri Milan, Italy and authenticated by STR method) were grown in DMEM medium (Thermo Fisher Scientific, Milano, Italy), supplemented with 115 units/mL of penicillin G (Thermo Fisher Scientific, Milano, Italy), 115 µg/mL of streptomycin (Thermo Fisher Scientific, Milano, Italy) and 10% fetal bovine serum (Thermo Fisher Scientific, Milano, Italy). Cells were maintained at 37 °C in a humidified 5% CO_2_ incubator. The cells were periodically checked for mycoplasma contamination. No cell lines used in this paper were listed in the database of commonly misidentified cell lines maintained by International Cell Line Authentication Committee.

### Flow cytometry

Cells were treated with ANANAS formulations (Supplementary Table 2) or antibody conjugates. After the incubation period, cells were collected, centrifuged and analyzed on a Cytomic FC500 flow cytometer (Beckman Coulter). EGFR expression on cell surface was evaluated by staining cells with 0.5 or 1 µg/mL of Cetux-Atto488 for 30 min. Data are presented as median fluorescence intensity of positive cells.

### Immunoflorescence analysis

After treatment, cells were incubated for 45 min with Lysotracker Deep Red (1:1000, Life technologies), fixed in cold 4% formaldehyde for 15 min, rinsed and stored prior to analysis.

Cell nuclei were counterstained with DAPI (1:10000, Sigma-Aldrich). Images were obtained on a video-confocal microscope (Vico, Ecliple Ti80, Nikon), equipped with a digital camera.

### Cell proliferation in vitro (MTT test)

Cells were treated for 6 h with ANANAS formulations, washed out and incubated with normal medium for additional 72 h. Cell proliferation was assessed by the 3-(4,5-dimethylthiazol-2-yl)-2,5-diphenyl tetrazolium bromide (MTT) test^43^.

### Live fluorescence microscopy

Live-cell fluorescence imaging was performed (see also Supplementary Method 2) using a IX83 Inverted Microscope (Olympus) equipped with ORCA-Flash4.0camera (Hamamatsu), PLAPON 60xOPh/1.4 objective (Olympus), hard coated ET type filters 49000, 49002, 49008, 49009 (Chroma), and Lumen 1600 light source (Prior). Images were processed using cellSens Dimension (Olympus) and ImageJ (NIH).

Human MDA-MB-231 or MCF-7 (4 × 104) cells were grown on an μ–Slide VI0.4 ibiTreat (ibidi). A day later, the cells were labeled with 20 µM Hoechst 33342 for 5 min and the ibidi slide was connected to a microfluidic flow system. ANANAS nanoparticles were perfused over the cells in binding medium (Hank’s balanced-salt solution containing 2 mg/mL bovine serum albumin and 10 mM 4-(2-hydroxyethyl)-1-piperazineethanesulfonic acid, pH 7.4, supplemented with CaCl_2_, MgCl_2_) for up to 20 min at low flow rate (0.1 ml/min). Cells were subsequently subjected to continuous flow (a sub-physiological shear stress of 0.02 dyn/cm^2^) for the rest of the assay in order to maintain them in a nutrient rich environment and remove unbound material. Images were acquired at a rate of 1 frame every 2 min for the first hour or 1 frame every 6 min for 6 h.

### In vivo antitumor assay

Animal experiments were approved by the local animal ethics committee (OPBA, Organismo Preposto al Benessere degli Animali, Università degli Studi di Brescia, Italy) and were executed in accordance with national guidelines and regulations. Procedures involving animals and their care conformed with institutional guidelines that comply with national and international laws and policies (EEC Council Directive 86/609, OJ L 358, 12 December 1987) and with “ARRIVE” guidelines (Animals in Research Reporting In Vivo Experiments).

Human MDA-MB-231 (5 × 10^6^) breast cancer cells were injected subcutaneously in the flank of 6-week-old NOD/SCID female mice. Two weeks post implantation, when tumors were visible, animals were randomized and treated every other day (total four doses) by i.v. injection with ANANAS-doxo-cetux10 (0.56 mg/kg in doxorubicin and 2.8 mg/kg in cetuximab) or control formulations (ANANAS-doxo (0.56 mg/kg in doxorubicin), cetuximab (2.8 mg/kg), doxorubicin (at 5 or 0.56 mg/kg)) in 100 µl final volume. Cetux-doxo (total four doses, each 0.56 mg/kg in doxorubicin, 90 mg/kg in cetuximab) was administered via i.p. in 350 µl volume. Tumors were measured in two dimensions and tumor volume was calculated according to the formula *V* = (*D* × *d*^2^)/2, where *D* and *d* are the major and minor perpendicular tumor diameters in mm, respectively At the end of the experimental procedure tumors were harvested and embedded in OCT and frozen for histological processing.

### Immunohistochemistry and immunofluorescence of tumor sections

Excised tumors were cut with a cryostat in 4–5 µm sections. Immunohistochemistry was performed by staining samples with anti-Ki67 antibody (1:100, Dako, # M7240; clone MIB-1; lot. 20014345) and biotinylated horse anti-mouse secondary antibody (1:100, VECTASTAIN ABC kit, Vector Laboratories). Hematoxylin and eosin was also performed to visualize the histological features of tumors. Apoptotic nuclei were evaluated by using Tunel technology, following the In Situ Cell Death Detection Kit (Fluorescein, Sigma-Aldrich) instructions. All specimens were viewed under a video-confocal microscope (Zeiss Axio Imager M1), equipped with a digital camera, and images were captured using a × 20 or × 40 objective (see also Supplementary Method 2).

### Statistical analysis

Graph Pad Prism 6.07 (GraphPad, La Jolla, CA) was used to generate graphs and associated statistical analyses. All data are presented as mean ± standard error or standard deviation. Statistical significance was measured by paired *t* test in experiments performed in the same cell line, whereas comparison between different cell lines was evaluated by unpaired *t* test; *p < 0.05, **p < 0.01, ***p < 0.001, ****p < 0.0001. Tumor volume data were statistically analyzed with a two-way analysis of variance, and individual group comparisons were evaluated by the Bonferroni correction. Differences were considered significant when *P* < 0.05.

## Electronic supplementary material

Supplementary Movie 1

Supplementary Movie 2

Supplementary Movie 3

Supplementary Movie 4

Supplementary Movie 5

Supplementary Movie 6

Supplementary Information

Description of Additional Supplementary Files

## Data Availability

Relevant data generated during and/or analyzed during the current study are available from the corresponding author on reasonable request.
