# Supplementary material for: Improvement and extension of anti-EGFR targeting in breast cancer therapy by integration with the Avidin-Nucleic-Acid-Nano-Assemblies
Source: Nat Commun. 2018 Oct 4;9:4070. doi: 10.1038/s41467-018-06602-6 (PMC6172284; doi:10.1038/s41467-018-06602-6)
Supplement: Supplementary file 7 — Supplementary Information [file 41467_2018_6602_MOESM7_ESM.pdf]

# Improvement and extension of anti-EGFR targeting in breast cancer therapy by integration with the Avidin-Nucleic-Acid-Nano Assemblies

Roncato F. et al.

## Supplementary information

Supplementary Figures ........................................................................................................2
Supplementary Tables........................................................................................................14
Supplementary Methods......................................................................................................20
Supplementary References ..................................................................................................24

## Supplementary Figures

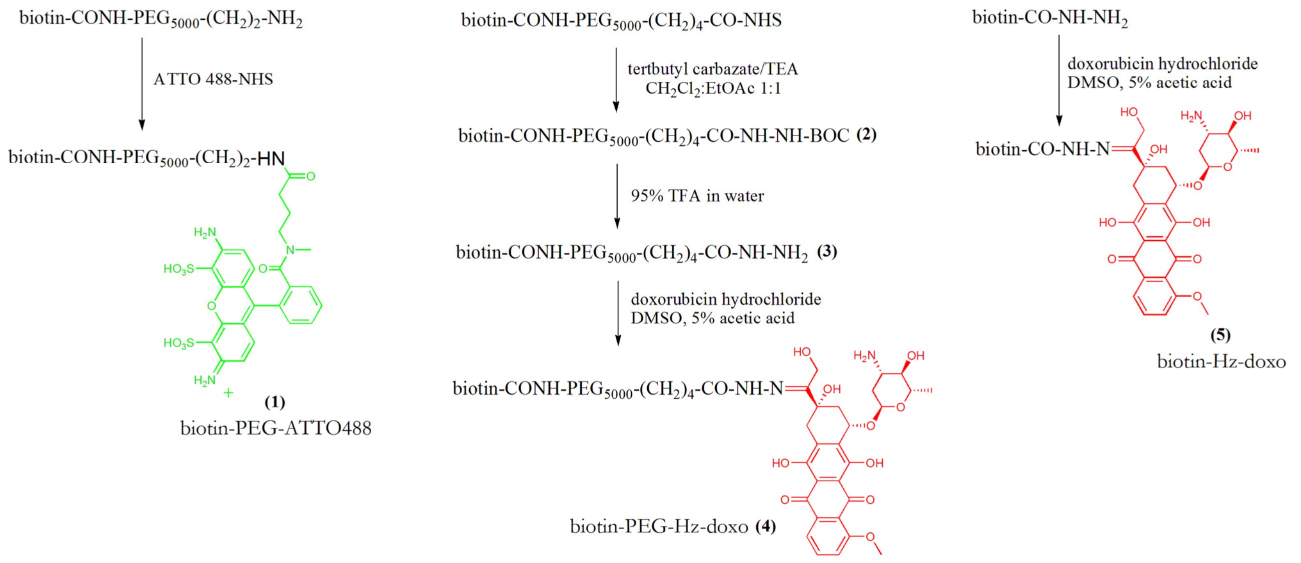


**Supplementary Figure 1**. *Synthetic steps in the preparation of the biotin doxorubicin and biotin Atto488 conjugates*

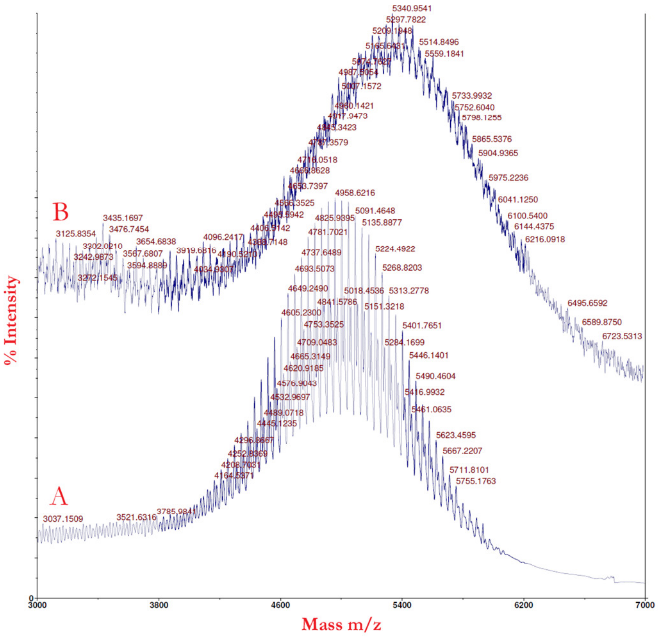


**Supplementary Figure 2.** *MALDI-TOF spectra* of (upper panel) biotin-PEG hydrazide (**c**ompound **3,**) and (lower panel) biotin-PEG-doxo (compound **4**); positive ion mode. The difference in average m/z between the two compounds is compatible with doxorubicin MW (544 Da).

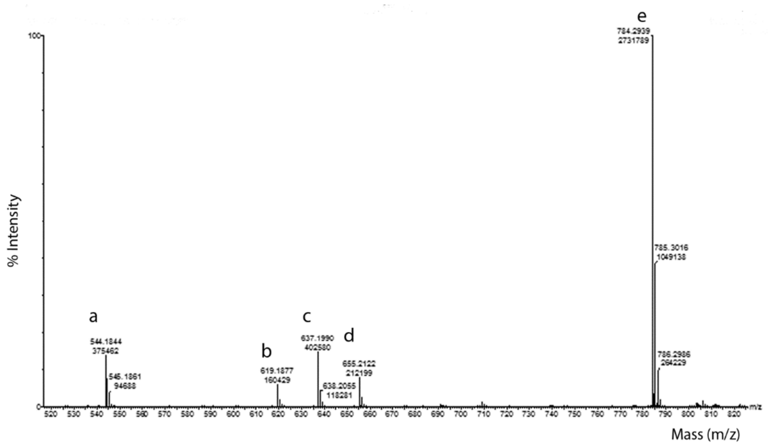


**Supplementary Figure 3.** *ESI TOF mass spectrum of biotin-Hz-doxo* (compound **5**). Mass (m/z) values for each peak: a) 544.1844 b) 619.1877 c) 637.1990 d) 655.2122 e) 784.2393. Peak a) corresponds to free doxorubicin; e) to compound 5 while peaks b), c), d) to the biotin hydrazine of doxorubicin aglycons.

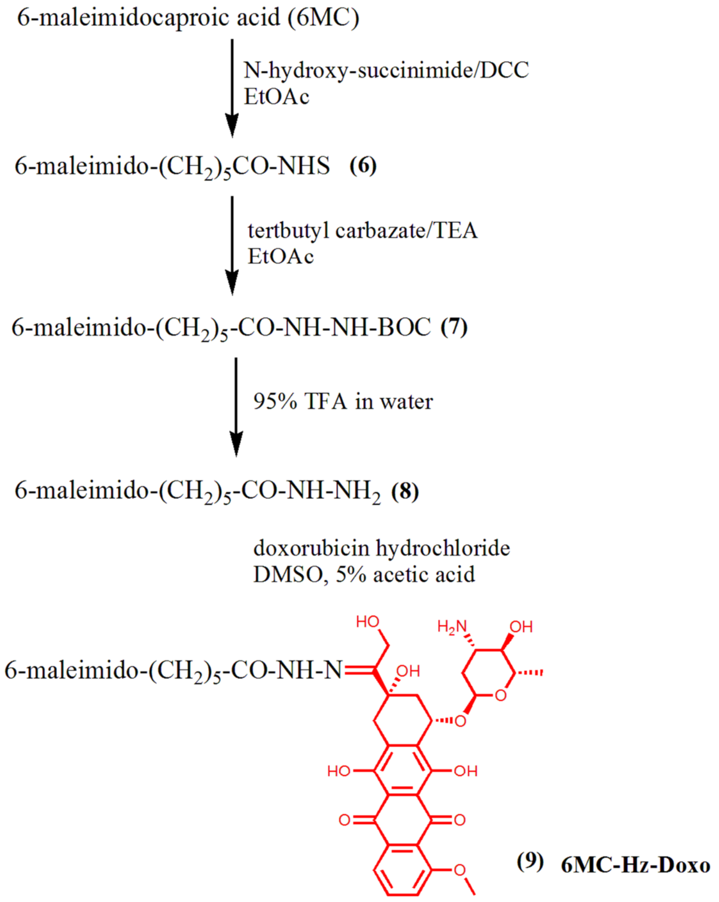


**Supplementary Figure 4.** *Synthetic steps followed for the preparation of doxorubicin 6-Maleimidocaproic hydrazone (6MC-Hz-doxo)*

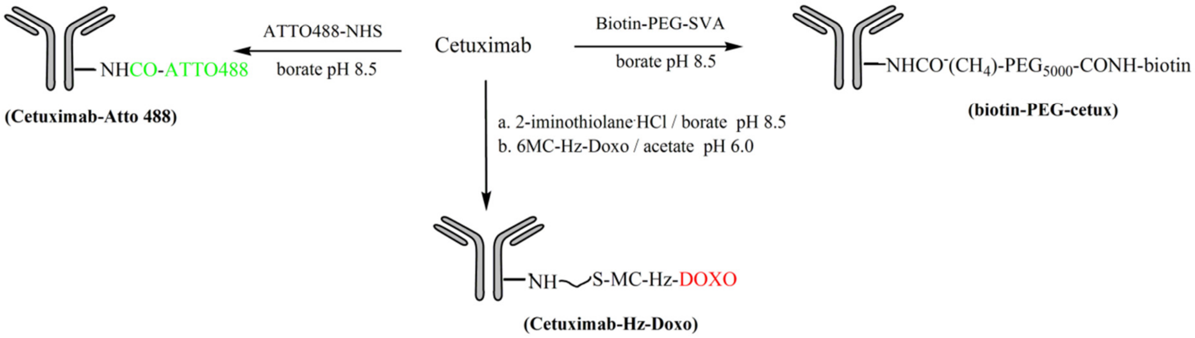


**Supplementary Figure 5.** *Synthetic steps followed for the preparation of cetuximab conjugates.*

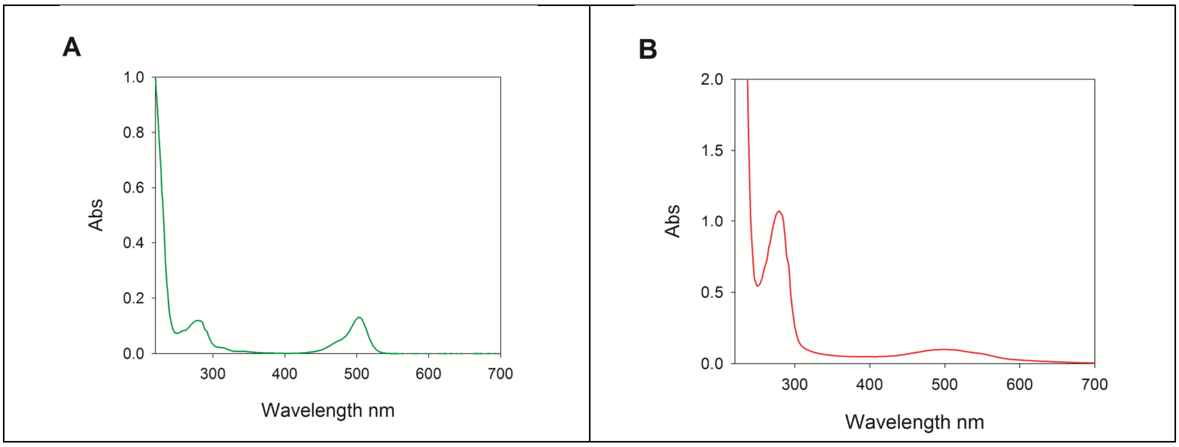


**Supplementary Figure 6.** *UV-Vis spectra* of Cetuximab Atto488 (panel A) and Cetuximab doxorubicin conjugate (panel B). The antibody to drug/dye ratios were determined from these spectra taking into consideration the component absorptivities (Atto488 $\varepsilon_{501nm} = 9x10^4$; Doxo $\varepsilon_{480nm} = 11500$; IgG $E_{280nm\ 0.1\%,\ 1cm} = 1.45$ ) and attested at 1.4 for Cetux-ATTO488 and at 1.8 for Cetux-doxo. The UV-vis spectra recorded in PBS are shown below.

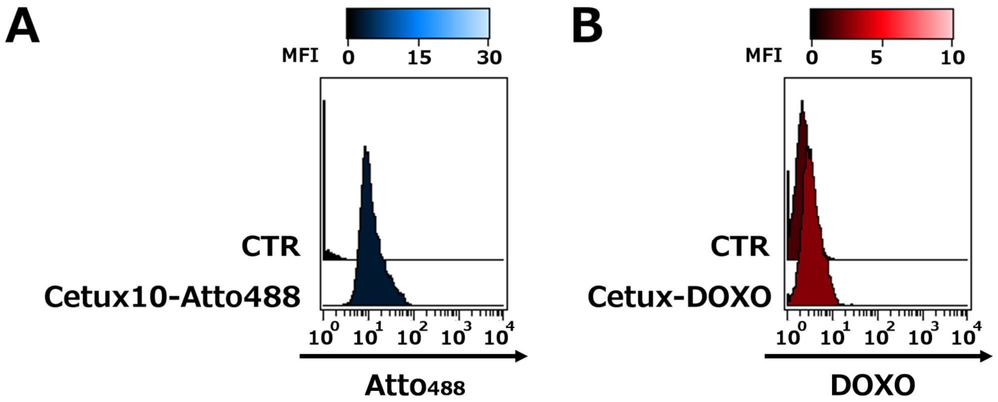


**Supplementary Figure 7** . *Functional evaluation of cetuximab Atto488 and doxorubicin conjugates*. The ability of the modified antibody to recognize its target antigen was tested by Flow Cytometry (FACS). Representative flow cytometry histograms showing Cetux10-Atto488 (panel A) or Cetux-DOXO (panel B) related signals in MDA-MB-231 cells incubated for 6 hours with the antibody conjugates at a concentration of 7.5 µg/ml. The upper histogram in each panel shows the control signal generated by untreated cells. Color scale indicates mean fluorescence intensity (MFI) values. A positive shift in MFI indicates the ability of the cetuximab conjugate to recognize the cell EGFR.

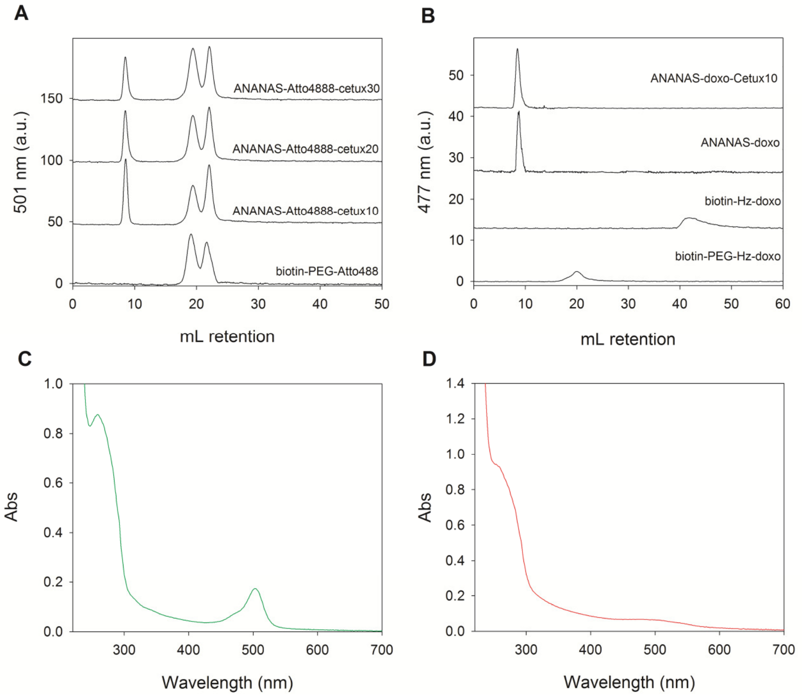


**Supplementary Figure 8** *Analysis of ATTO488-labelled and doxorubicin-loaded ANANAS formulations.* **A)** Size-exclusion *(Superose Prep 30/100 GL column)* elution profiles of ANANAS-Cetuximab at three Ab/NP molar ratios: 10, 20, 30) labelled with biotin-PEG-ATTO488 at a 30% BBS coverage. biotin-PEG-ATTO488 was analysed at the same concentrations as used for ANANAS loading. The products eluting at 8.5 mL correspond to the ANANAS-Cetuximab-ATTO488, the one at 19.5 mL is the biotin-PEG-ATTO488 conjugate while the peak at 22.1 mL corresponds to the free ATTO488 molecule, which was present as an impurity in the biotin-PEG-Atto488 product. Chromatograms were analysed at 501 nm. **B)** ANANAS-doxo and ANANAS-Cetux10-doxo size-exclusion *(Superose Prep 30/100 GL column)* elution profiles show the quantitative binding to ANANAS of both biotin-PEG-Hz-doxo (at 30% BBS occupancy) and biotin-Hz-doxo (at 40% BBS occupancy). Chromatograms were analysed at 477 nm. **C)** UV-vis spectrum of FPLC-purified ANANAS-ATTO488-cetux10 formulation at 200 µg/ml. **D)** UV-vis spectrum of ANANAS-doxo-cetux10 at 200 µg/ml.

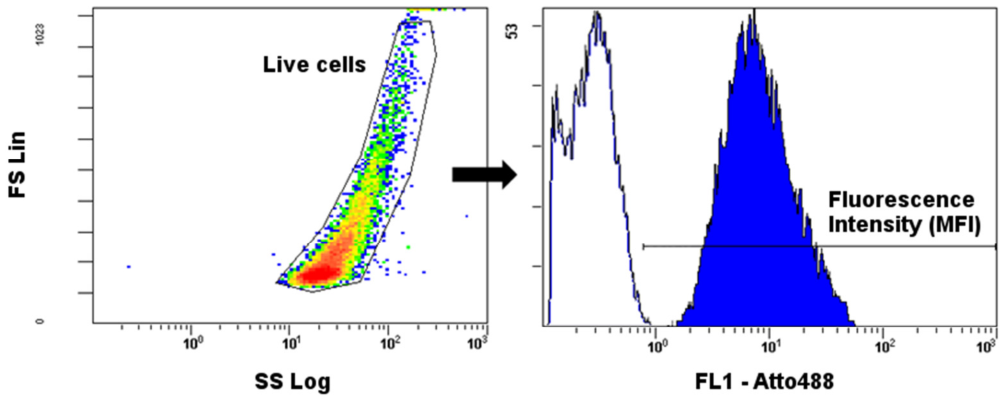


**Supplementary Figure 9.** *Gating strategy* used for the flow cytometry analyses presented in Main text Fig. 2 and Fig. 3A,B,C,E.

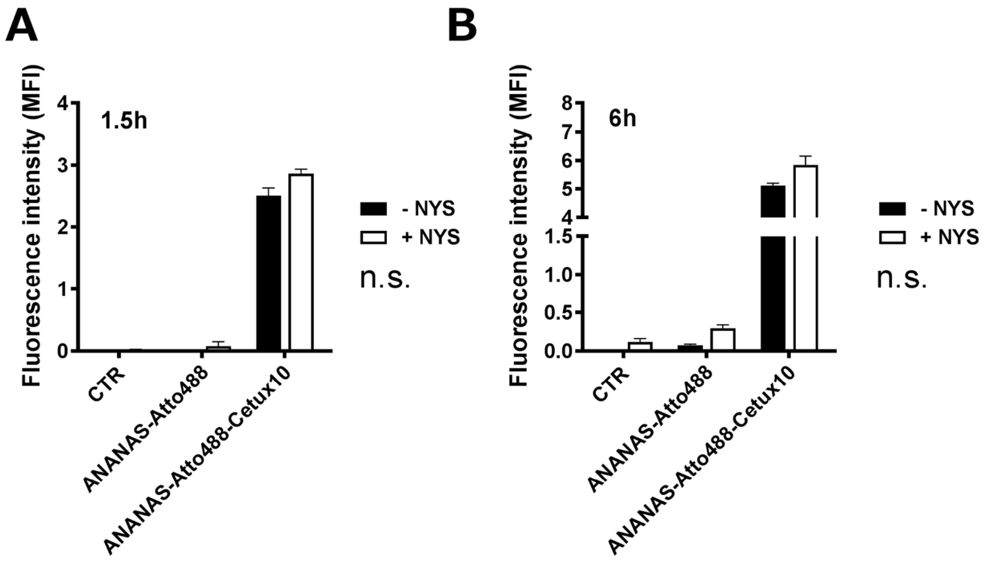


**Supplementary Figure 10** *ANANAS internalization in the presence of nystatin*. Median fluorescence intensity (MFI) associated with MDA-MB-231 cells pre-treated with nystatin 50 µg/mL for 30 minutes, and then treated with for 1.5 (A) and 6 h (B) with the different formulations. Data are presented as mean of n=3 independent experiments, each performed in triplicates ± SEM. For the concentration of Atto 488 loaded reagents, see figure 3 in the manuscript.

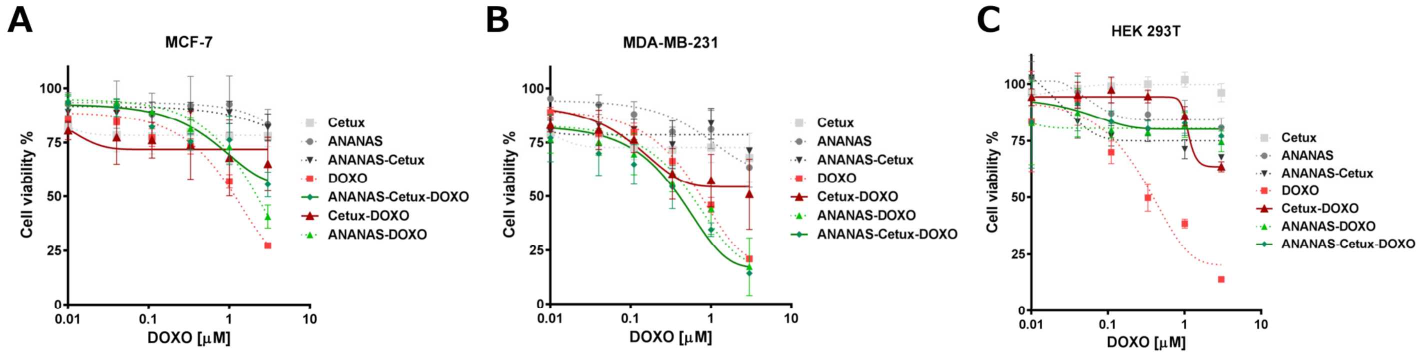


**Supplementary Figure 11.** *In vitro activity of Doxo loaded formulations*. Cell viability was assessed in the indicated cell lines (two breast cancer cell lines MCF 7, MDA-MB-231 and normal human epithelial cell HEK-293 after 6 h treatment and wash out for following 72 h. Cell viability was measured by MTT test. Data presented as mean of two experiments, each performed in triplicates ± SEM.

.

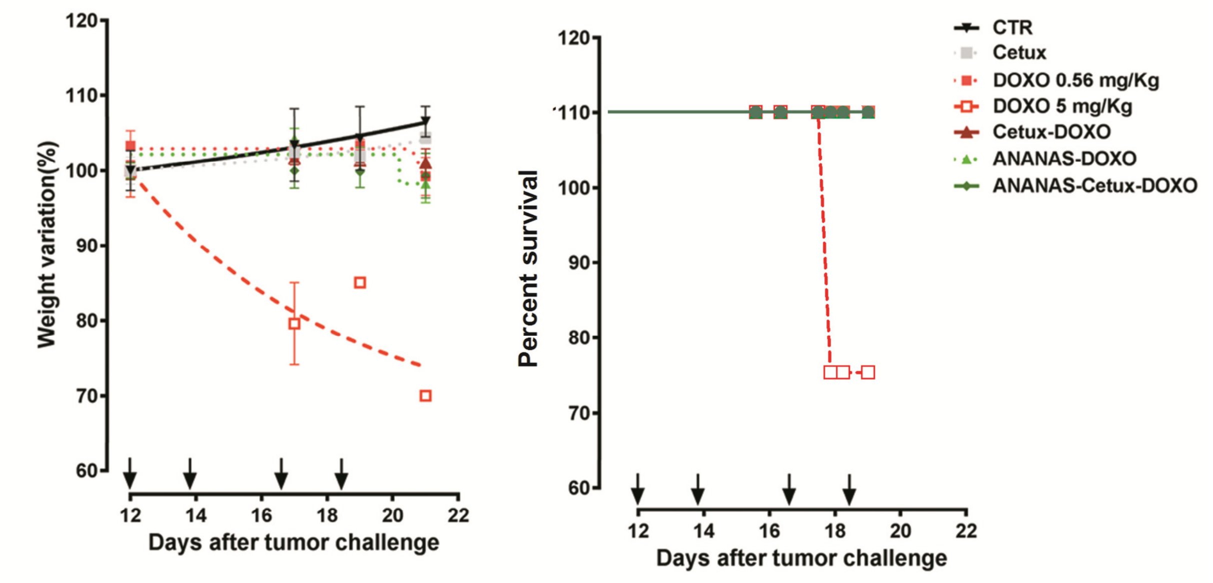


**Supplementary Figure 12.** *In vivo toxicity of different doxorubicin formulation treatments.* **A)** Animal (n=6/group) weight variations during the course of treatment with ANANAS formulations. The arrows indicate time of administration; **B)** Animal survival curves. Data are presented as mean ± SD.

# Supplementary Tables

**Supplementary Table 1.** Physico-chemical properties *of Atto488 carrying formulations.*

| Formulation name | Cetuximab/NP (mole: mole) | Atto488/NP (mole: mole) | FAR(*) | Atto488 brilliance % conjugated vs free | Size Z-average (nm) (**) | PdI (***) | ζ pot (mV) (****) |
|---|---|---|---|---|---|---|---|
| **ANANAS-Atto488-cetux 10** | 10 | 230 | 23.0 | 40% | 143.4±0.3 | 0.22±0.00 | -7.87±0.56 |
| **ANANAS-Atto488-cetux 20** | 20 | 196 | 9.8 | 40% | 140.5±0.8 | 0.21±0.00 | -6.67±0.54 |
| **ANANAS-Atto488-cetux 30** | 30 | 161 | 5.4 | 40% | 140.9±0.3 | 0.21±0.01 | -4.02±0.24 |
| **ANANAS-Atto488** | 0 | 270 | - | 40% | 135.7±4.5 | 0.26±0.05 | -11.47±0.32 |
| **Cetux-Atto488** | - | - | 1.4 | 61% | | | |

**(*) FAR:** Fluorophore to Antibody Ratio
(**) Size data are displayed as the mean of three measurements ± SD
(***) PdI: Poly Dispersivity: the value is the mean of three measurements ± SD
(****) ζ pot values are displayed as the mean of three measurements ± SD

**Supplementary Table 2.** *Physico-chemical properties doxorubicin carrying formulation.*

| Formulation name | Cetuximab/ NP (mole: mole) | B-PEG (*) (B-PEG-Doxo) /NP (mole: mole) | B-EZ (**) (B-Ez-Doxo) /NP (mole: mole) | DAR (***) | Size, Z-average (nm) (****) | PdI (*****) | ζ pot (mV) (******) |
|---|---|---|---|---|---|---|---|
| **ANANAS-doxo-cetux 10** | 10 | 500(177) | 536(472) | 94(65) | 149.8±3.6 | 0.22 ±0.02 | -7.51 ±0.85 |
| **ANANAS-doxo** | 0 | 570(177) | 536(472) | - | 131.6±3.1 | 0.22 ±0.03 | -11.70 ±2.69 |
| **Cetux-doxo** | - | - | - | 1.8 | - | | - |

(*) The total BPEG number comprises the biotin-PEG chains of the core ANANAS formulation, the fraction of unconjugated biotin-PEG-hydrazine and the doxorubicin hydrazone conjugate

(**) The total biotin-EZ number comprises the unconjugated biotin-EZ-hydrazine and the doxorubicin hydrazone conjugate.

(***) DAR**:** Drug (doxorubicin) to Antibody Ratio

(****) Size data are displayed as the mean of three measurements ± SD

(*****) PdI: Poly Dispersivity: the value is the mean of three measurements ± SD

(******) ζ pot values are displayed as the mean of three measurements ± SD

**Supplementary Table 3.** *Doxorubicin release kinetics.*

| | Release % / hour | half-life (h) | T-ex |
|---|---|---|---|
| **Biotin-Hz-Doxo** | 5.7+/-0.95 | 8.8+/-1.2 | 17.6+/-2.5 |
| **Biotin-Hz-Doxo+ FCS** | 5.1+/-0.4 | 9.7+/-0.8 | 19.5+/-1.5 |
| **Biotin-Hz-Doxo + ANANAS** | 1.8+/-0.04 | 28.2+/-0.6 | 56.5+/-1.2 |
| **Biotin PEG-Hz-Doxo** | 7.0+/-0.4 | 7.1+/-0.4 | 14.2+/-0.8 |
| **Biotin PEG-Hz-Doxo + ANANAS** | 6.0+/-0.2 | 8.4+/-0.2 | 16.7+/-0.5 |

(NOTE) No release was observed at pH 7.0, in the presence or absence of fetal calf serum (FCS).

Doxorubicin release/hour from the different Doxorubicin hydrazone conjugates, as free molecules or when tethered to the ANANAS, measure at pH 5.0 in the presence or absence of fetal calf serum (FCS). Release values are expressed as percentage over total. Release experiments were carried out in duplicates and data are presented as the mean ± S.D.

**Supplementary Table 4.** *Relative EGFR expression in MCF7 and MDA-MB-231***.**

| µg/mL Cetux-atto488 | MCF7 | MDA-MB231 |
|---|---|---|
| **1** | 0.015+/-0.008 | 0.34+/-0.10 |
| **0.5** | 0.015+/-0.011 | 0.28+/-0.06 |

Median fluorescence intensity (n=3) associated with MCF7 or MDA-MB-231 cells after 30 min incubation with 0.5 or 1 µg/mL of cetuximab-Atto488. Data presented as the mean of three measurements (single experiment carried out in triplicate) ± SEM.

**Supplementary Table 5.**
*Median fluorescence intensity* associated with cells upon treatment (6h) with different Atto488 formulations, in the presence or absence of chlorpromazine.

| Formulation name (*) | MCF7 | MCF7 + CPZ | MDA | MDA +CPZ |
|---|---|---|---|---|
| ANANAS-Atto488 | 0.44+/-0.12 | 0.37+/-0.13 | 0.15+/-0.15 | 0.12+/-0.16 |
| ANANAS-Atto488-cetux10 | 1.72+/-0.26 | 1.37+/-0.15 | 22.46+/-6.72 | 10.4+/-1.48 |
| Cetux-Atto488 (1) | 0.01+/-0.01 | 0.00+/-0.02 | 0.62+/-0.05 | 0.58+/-0.03 |
| Cetux-Atto488 (2) | 0.16+/-0.1 | 0.12+/-0.05 | 0.77+/-0.04 | 0.61+/-0.07 |

(*) Concentrations used in the experiment: ANANAS-Atto488: Atto 488 $3.5x10^{-9}$ M; ANANAS-Atto488-cetux10: cetuximab 0.58 μg/mL, Atto 488 $3.5x10^{-9}$M; cetux-Atto488(1): same cetuximab as in ANANAS-Atto488-cetux; cetux-atto488(2): same Atto488 as in ANANAS-Atto488-cetux; Data presented as mean of 3 independent experiments (each in triplicate) ± SEM.

**Supplementary Table 6.** *Ratio between cell-associated fluorescence upon treatment with ANANAS-Atto488-cetux10 or Cetux-Atto488.*

| Cell type | 1h | 3h | 6h |
|---|---|---|---|
| **MCF7** | nd | 42.73+/-7.45 | 139+/-41.14 |
| **MDA-MB-231** | 27.48+/-1.35 | 38.67+/-7.64 | 45.31+/7.38 |

***Notes***
*Cells were exposed to the same cetuximab concentration (0.58 µg/mL). For calculation, the mean fluorescence intensity (MFI) values were been normalized for the relative Atto488 brilliance when tethered to either the NP core or the antibody surface; FAR ANANAS-Atto488-cetux/FAR Cetux-Atto488 = 16.9, Supplementary Table 1)*
*Data presented as mean of n=3 experiments ± SEM*

## Supplementary Methods

**Supplementary Method 1.** *Synthesis and characterization of doxorubicin 6-maleimidocaproic hydrazone (6MC-Hz-doxo, compound 9)*

This compound was synthesized aas summarized in Supplementary Figure 4, following a protocol adapted from the literature (1). 10 mmoles of 6MC were dissolved with 11 mmoles of NHS in dry EtOAc; once the solution was clear, 11 mmoles of DCCI were added, leaving the flask under ice for the first 30' minutes. The reaction mixture was then left stirring at RT overnight. The trend of the reaction was monitored by TLC (90:10 $CH_2Cl_2$:MeOH mobile phase, RFs: 6MC = 0.32; NHS = 0.35; product 6MC-NHS = 0.81). The solution was added of 6 drops of glacial acetic acid and filtered for eliminating the DCU (dicyclohexylurea); the solvent was evaporated under vacuum and the residual oil was treated with hot *i*-PrOH where it dissolved rather fast, and left overnight at 0°C. The next day supernatant *i*-PrOH was removed and the semisolid mass was thoroughly worked out with $Et_2O$ which was likewise removed and dried separately, leaving a white crystalline powder. Quantitative NHS activation was confirmed by $^1$H-NMR analysis
($^1$H NMR (CDCl3, 300MHz) of compound 6 (**Supplementary Figure 5**): δ6.68 ppm (s, 2H, -C<u>H</u>=C<u>H</u>-, in maleimide ring). δ2.83 ppm (s, 4H, -C<u>H</u>$_2$-C<u>H</u>$_2$-, in NHS ring). Reaction yield: 30%
6MC-NHS (compound **6)** was added of 1.2 equiv. of boc-Hz and 2 equiv. of TEA in dry EtOAc; After overnight reaction at room temperature, the mixture was washed with 0.1 HCl M, and saturated $NaHCO_3$ aqueous solution (3 times each), then dried over $Na_2SO_4$ and concentrated under vacuum. $^1$H-NMR of the oily product confirmed full conversion into the desired 6MC-HzBOC.
($^1$H NMR (CDCl3, 300MHz) of compound 7: δ6.68 ppm (s, 2H, -C<u>H</u>=C<u>H</u>-, in maleimide ring). δ1.46 ppm (s, 3H, -O-(CH3)$_3$-, BOC group)
Removal of the BOC protecting group was obtained upon treatment with 95% TFA (30'). After elimination of TFA under vacuum, complete BOC removal was confirmed by $^1$H-NMR (NMR)
6MC-Hz (compound 9, **Supplementary Figure 5**) was mixed with 3-fold molar excess of Doxorubicin hydrochloride (final doxorubicin concentration 60 mg/mL) in 5% CH3COOH in dry DMSO. The reaction was left at RT for 24h and was directly used for the conjugation with the monoclonal antibody cetuximab. The protocol was adapted from the literature (2).

**Supplementary Method 2.** *Equipment and settings*

***FIGURE 3.***

Nikon Vico eclipse 80i microscope was used equipped with: Plan Apo VC 60x/1.40 Oil (∞/0.17 WD 0.13) objective; 31000V2 DAPI C75005 filter (DAPI, Ex. 364 nm, Em. 454 nm), FITC EX465-495 DM505 BA 515-555 filter (Atto488, Ex. 500 nm, Em. 520 nm), TRITC EX540/25 DM565 BA605/55 filter (LysoTracker Deep Red, Em. 647 nm, Em. 668); Q IMAGING QICAM FAST1394 camera. The Image-Pro PLUS version 6.0.0.260 software was used. Space resolution data (xy and pixel dimensions): Size (pixel) of each image: 1392 x 1040, Pixel dimensions: 232.56 x 232.56 nm, Image bit depth: 16-bit.Scale bar 20 µm.

***FIGURE 4.***

Olympus IX83 Inverted Microscope equipped with: Olympus Objective PLAPON 60xOPh/1.4 and Chroma filters: filter set 49002 - ET - EGFP (FITC/Cy2) and filter set 49000 - ET – DAPI; Camera:ORCA-Flash4.0LT camera (Hamamatsu) Model: C11440-42U; Software Olympus cellSens Dimension version 1.16 for acquisition and manipulation (background subtraction in both the fluorescent channels used); Space resolution data (xy and pixel dimensions): Size (pixel) of each image in the grid: 966 x 970, Pixel dimension: 107.88 x 107.88 nm, Image bit depth: 24-bit; Fluorochrome Atto488 (ATTO-TEC GmbH), Absorption 500 nm, emission 520 nm; Fluorochrome: Hoechst 33342 (Thermo Scientific), absorption 350 nm, emission 461 nm. Imaging medium: Hank's balanced-salt solution containing 2 mg/mL BSA and 10 mM HEPES, pH 7.4, supplemented with CaCl2, MgCl2, Temperature: 37 ºC, Scale bar 20 µm.

***FIGURE 5.***

Olympus IX83 Inverted Microscope equipped with: Olympus Objective PLAPON 60xOPh/1.4 and Chroma filters: filter set 49008 - ET - mCherry, Texas Red®, and filter set 49000 - ET - DAPI Camera:ORCA-Flash4.0LT camera (Hamamatsu) Model: C11440-42U; Software Olympus cellSens Dimension version 1.16 for acquisition and manipulation (background subtraction in both the fluorescent channels used); Space resolution data (xy and pixel dimensions): Size (pixel) of each image in the grid: 1278 x 835, Pixel dimension: 107.88 x 107.88 nm, Image bit depth: 24-bit; Fluorochrome Doxorubicin (Sigma-Aldrich): absorption 550 nm, emission 578 nm; Fluorochrome: Hoechst 33342 (Thermo Scientific), absorption 350 nm, emission 461 nm. Imaging medium: Hank's balanced-salt solution containing 2 mg/mL BSA and 10 mM HEPES, pH 7.4, supplemented with $CaCl_2$, $MgCl_2$, Temperature: 37 ºC, Scale bar 20 µm.

***FIGURE 6.***

Zeiss Axio Imager M1 microscope equipped with EC Plan-Neofluar 40x/0.75 M27; filters: DAPI 424920 filter (DAPI, Ex. 364 nm, Em. 454 nm), FITC 424920 Filterset10 filter (TUNEL, Ex. 450-500 nm, Em. 515-565 nm), Brightfield filter; AxioCam MRc5 ZEISS camera. The AxioVs40x64 V 4.9.1.0 software was used. Space resolution data (xy and pixel dimensions): Size (pixel) of each image: 1499 x 1123, Pixel dimensions: 166.67 x 166.67 nm, Image bit depth: 32-bit. Scale bar 25 µm.

*Supplementary Movie 1.*

IX83 Inverted Microscope (Olympus) equipped with ORCA-Flash4.0camera (Hamamatsu), PLAPON 60xOPh/1.4 objective (Olympus), hard coated ET type filters 49000, 49002, 49008, 49009 (Chroma) and Lumen 1600 light source (Prior). For image acquisition, the microscope was used with Chroma 49000 filter set for Hoechst 33342 (Blue, Ex. 365 nm; Em. 461 nm) and 49002 filter set for ATTO488 (Green, Ex. 500 nm; Em. 520 nm). Calibration (X): 107.88 nm/pixel. Calibration (Y): 107.88 nm/pixel. Size (pixel): 1920 x 960. Time-lapse interval: 00:02:00 (2 minutes). Frame rate: 4 fps. Scale bar: 20 µm. Background subtraction was applied to both the fluorescent channels using cellSens Dimension software 1.16 (Olympus). ImageJ (NIH) software was used for title and time code labeling and for combining together the stacks of the original videos.. Imaging medium: Hank's balanced-salt solution containing 2 mg/mL BSA and 10 mM HEPES, pH 7.4, supplemented with $CaCl_2$, $MgCl_2$. Temperature: 37 ℃

*Supplementary Movie 2.*

IX83 Inverted Microscope (Olympus) equipped with ORCA-Flash4.0camera (Hamamatsu), PLAPON 60xOPh/1.4 objective (Olympus), hard coated ET type filters 49000, 49002, 49008, 49009 (Chroma) and Lumen 1600 light source (Prior). For image acquisition, the microscope was used with Chroma 49000 filter set for Hoechst 33342 (Blue, Ex. 365 nm; Em. 461 nm) and 49002 filter set for ATTO488 (Green, Ex. 500 nm; Em. 520 nm). Calibration (X): 107.88 nm/pixel. Calibration (Y): 107.88 nm/pixel. Size (pixel): 960 x 823. Time-lapse interval: 00:02:00 (2 minutes). Frame rate: 4 fps. Scale bar: 20 µm. Background subtraction was applied to both the fluorescent channels using cellSens Dimension software 1.16 (Olympus). ImageJ (NIH) software was used for title and time code labeling.. Imaging medium: Hank's balanced-salt solution containing 2 mg/mL BSA and 10 mM HEPES, pH 7.4, supplemented with $CaCl_2$, $MgCl_2$. Temperature: 37 ℃

*Supplementary Movie 3.*

IX83 Inverted Microscope (Olympus) equipped with ORCA-Flash4.0camera (Hamamatsu), PLAPON 60xOPh/1.4 objective (Olympus), hard coated ET type filters 49000, 49002, 49008, 49009 (Chroma) and Lumen 1600 light source (Prior). For image acquisition, the microscope was used with Chroma 49000 filter set for Hoechst 33342 (Blue, Ex. 365 nm; Em. 461 nm) and 49002 filter set for ATTO488 (Green, Ex. 500 nm; Em. 520 nm). Calibration (X): 107.88 nm/pixel. Calibration (Y): 107.88 nm/pixel. Size (pixel): 1920 x 876. Time-lapse interval: 00:02:00 (2 minutes). Frame rate: 4 fps. Scale bar: 20 µm. Background subtraction was applied to both the fluorescent channels using cellSens Dimension software 1.16 (Olympus). ImageJ (NIH) software was used for title and time code labeling and for combining the stacks of the original videos. Imaging medium: Hank's balanced-salt solution containing 2 mg/mL BSA and 10 mM HEPES, pH 7.4, supplemented with $CaCl_2$, $MgCl_2$. Temperature: 37 ℃

*Supplementary Movie 4.*

IX83 Inverted Microscope (Olympus) equipped with ORCA-Flash4.0camera (Hamamatsu), PLAPON 60xOPh/1.4 objective (Olympus), hard coated ET type filters 49000, 49002, 49008, 49009 (Chroma) and Lumen 1600 light source (Prior). For image acquisition, the microscope was used with Chroma 49000 filter set for Hoechst 33342 (Blue, Ex. 365 nm; Em. 461 nm) and 49002

filter set for ATTO488 (Green, Ex. 500 nm; Em. 520 nm). Calibration (X): 107.88 nm/pixel. Calibration (Y): 107.88 nm/pixel.
Size (pixel): 960 x 885. Time-lapse interval: 00:06:00 (6 minutes). Frame rate: 10 fps. Scale bar: 20 µm. Background subtraction was applied to both the fluorescent channels using cellSens Dimension software 1.16 (Olympus). ImageJ (NIH) software was used for title and time code labeling.
. Imaging medium: Hank's balanced-salt solution containing 2 mg/mL BSA and 10 mM HEPES, pH 7.4, supplemented with $CaCl_2$, $MgCl_2$. Temperature: 37 ºC.

*Supplementary Movie 5.*

IX83 Inverted Microscope (Olympus) equipped with ORCA-Flash4.0camera (Hamamatsu), PLAPON 60xOPh/1.4 objective (Olympus), hard coated ET type filters 49000, 49002, 49008, 49009 (Chroma) and Lumen 1600 light source (Prior). For image acquisition, the microscope was used with Chroma 49000 filter set for Hoechst 33342 (Blue, Ex. 365 nm; Em. 461 nm) and 49002 filter set for ATTO488 (Green, Ex. 500 nm; Em. 520 nm). Calibration (X): 107.88 nm/pixel. Calibration (Y): 107.88 nm/pixel. Size (pixel): 1920 x 914. Time-lapse interval: 00:06:00 (6 minutes). Frame rate: 10 fps. Scale bar: 20 µm. Background subtraction was applied to both the fluorescent channels using cellSens Dimension software 1.16 (Olympus). ImageJ (NIH) software was used for title and time code labeling and for combining the stacks of the original videos.. Imaging medium: Hank's balanced-salt solution containing 2 mg/mL BSA and 10 mM HEPES, pH 7.4, supplemented with $CaCl_2$, $MgCl_2$. Temperature: 37 ºC.

*Supplementary Movie 6.*

IX83 Inverted Microscope (Olympus) equipped with ORCA-Flash4.0camera (Hamamatsu), PLAPON 60xOPh/1.4 objective (Olympus), hard coated ET type filters 49000, 49002, 49008, 49009 (Chroma) and Lumen 1600 light source (Prior). For image acquisition, the microscope was used with Chroma 49000 filter set for Hoechst 33342 (Blue, Ex. 365 nm; Em. 461 nm) and 49008 filter set for Doxorubicin (Red, Ex. 550 nm; Em. 578 nm). Calibration (X): 107.88 nm/pixel. Calibration (Y): 107.88 nm/pixel. Size (pixel): 1920 x 681. Time-lapse interval: 00:06:00 (6 minutes). Frame rate: 10 fps. Scale bar: 20 µm. Background subtraction was applied to both the fluorescent channels using cellSens Dimension software 1.16 (Olympus). ImageJ (NIH) software was used for title and time code labeling and for combining together the stacks of the original video and the red fluorescent channel.. Imaging medium: Hank's balanced-salt solution containing 2 mg/mL BSA and 10 mM HEPES, pH 7.4, supplemented with $CaCl_2$, $MgCl_2$. Temperature: 37 ºC.

## Supplementary References


(1) Keller, O. & Rudinger, J. Preparation and some properties of maleimido acids and maleoyl derivatives of peptides. Helvetica chimica acta 58, 531-541, doi:10.1002/hlca.19750580224 (1975).

(2) Willner, D. et al. (6-Maleimidocaproyl)hydrazone of doxorubicin--a new derivative for the preparation of immunoconjugates of doxorubicin. Bioconjugate chemistry 4, 521-527, doi: 10.1021/bc00024a015(1993).
