# Supplementary material for: Improvement and extension of anti-EGFR targeting in breast cancer therapy by integration with the Avidin-Nucleic-Acid-Nano-Assemblies
Source: Nat Commun. 2018 Oct 4;9:4070. doi: 10.1038/s41467-018-06602-6 (PMC6172284; doi:10.1038/s41467-018-06602-6)
Supplement: Supplementary file 8 — Description of Additional Supplementary Files [file 41467_2018_6602_MOESM8_ESM.docx]

**Title:** Supplementary Movie 1

**Description:** Live fluorescent imaging of ANANAS-Atto488-cetux10 (15 µg/mL) vs cetux-Atto488 (15.1 µg/mL) internalized by MDA-MB-231 cells. Cells are exposed to a flow carrying the fluorescent nanoparticles (t= 2-8 min) or antibody (t= 2- 10 min). Internal trafficking is displayed at t= 10-60 mins. Related to Figure 4.

**Title:** Supplementary Movie 2.

**Description:** Live fluorescent imaging of ANANAS-Atto488-cetux10 (15 µg/mL) internalized by MCF-7 cells. Cells are exposed to a flow carrying the nanoparticles at t= 2-8 mins. Internal particle trafficking is displayed at t= 10-60 mins. Related to Figure 4.

**Title:** Supplementary Movie 3.

**Description:** Live fluorescent imaging of ANANAS-Atto488 (15 µg/mL) over MDA-MB-231 and MCF-7 cells. Cells are exposed to a flow carrying the nanoparticles at t= 4-20 mins.

**Title:** Supplementary Movie 4.

**Description:** Live fluorescent imaging of Cetux-Atto488 (15.1 µg/mL) trafficking inside MDA-MB-231 cells after 1 hour from initial internalization with flow.

**Title:** Supplementary Movie 5.

**Description:** Live fluorescent imaging of ANANAS-Atto488-cetux10 (15 µg/mL), trafficking inside MDA-MB-231 and MCF-7 cells after 1 hour from initial internalization with flow.

**Title:** Supplementary Movie 6.

**Description:** Live fluorescent imaging of ANANAS-Hz-doxorubicincetux10 (35 µg/mL) trafficking inside MDA-MB-231 cells. Time-lapse video
